# Mining versatile feruloyl esterases: phylogenetic classification, structural features, and deep learning model

**DOI:** 10.1186/s40643-024-00835-8

**Published:** 2025-01-29

**Authors:** Liang Guo, Yuxin Dong, Deyong Zhang, Xinrong Pan, Xinjie Jin, Xinyu Yan, Yin Lu

**Affiliations:** 1 Key Laboratory of Pollution Exposure and Health Intervention of Zhejiang Province, College of Biological and Environment Engineering, Zhejiang Shuren University, Hangzhou, 310015 China; 2 Jinan No.1 High School, Jinan, 250014 China; 3 College of Life and Environmental Science, Wenzhou University, Wenzhou, Zhejiang 325035 China; 4 College of Agriculture, Yangtze University, Jingzhou, Hubei 434000 China

**Keywords:** Feruloyl esterases, Enzyme similarity network, Phylogenetic analysis, Molecular dynamics simulations, Deep learning

## Abstract

Feruloyl esterases (FEs, EC 3.1.1.73) play a crucial role in biological synthesis and metabolism. However, the identification of versatile FEs, capable of catalyzing a wide range of substrates, remains a challenge. In this study, we obtained 2085 FE sequences from the BRENDA database and initiated with an enzyme similarity network analysis, revealing three main clusters (1–3). Notably, both cluster 1 and cluster 3 included the characterized FEs, which exhibited significant differences in sequence length. Subsequent phylogenetic analysis of these clusters unveiled a correlation between phylogenetic classification and substrate promiscuity, and enzymes with broad substrate scope tended to locate within specific branches of the phylogenetic tree. Further, molecular dynamics simulations and dynamical cross-correlation matrix analysis were employed to explore structural dynamics differences between promiscuous and substrate-specific FEs. Finally, to expand the repertoire of versatile FEs, we employed deep learning models to predict potentially promiscuous enzymes and identified 38 and 75 potential versatile FEs from cluster 1 and cluster 3 with a probability score exceeding 90%. Our findings underscore the utility of integrating phylogenetic and structural features with deep learning approaches for mining versatile FEs, shedding light on unexplored enzymatic diversity and expanding the repertoire of biocatalysts for synthetic applications.

**Graphical Abstract:**

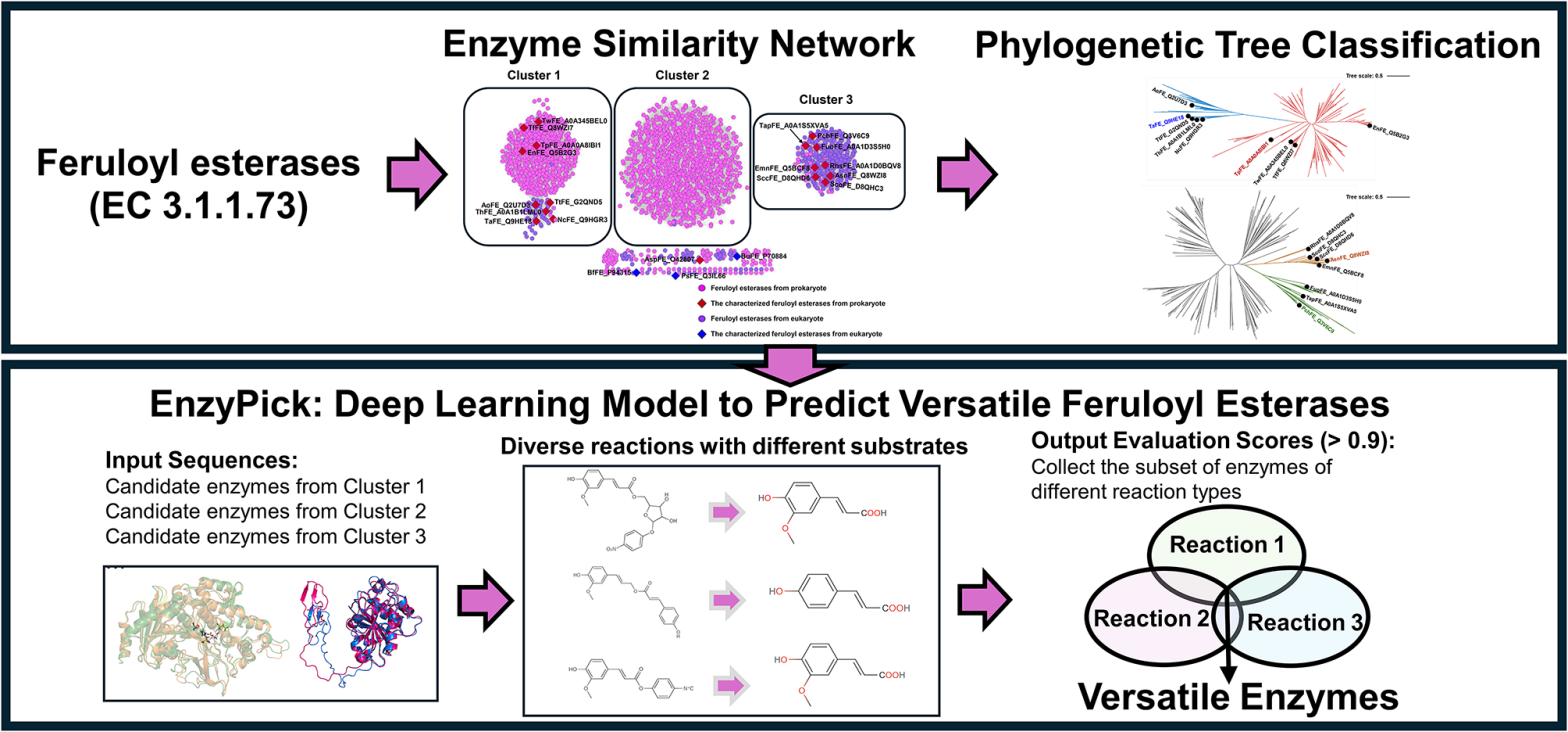

**Supplementary Information:**

The online version contains supplementary material available at 10.1186/s40643-024-00835-8.

## Introduction

Feruloyl esterases (FEs, EC 3.1.1.73) are pivotal enzymes with significant implications in various biological processes (Oliveira et al. 2019). These enzymes catalyze the hydrolysis of ester bonds between hydroxycinnamic acids and plant cell wall polysaccharides, contributing to the degradation of lignocellulosic biomass and the release of ferulic acid (Gopalan et al. 2015). Ferulic acid is a key aromatic compound widely used as a precursor in the synthesis of various valuable products, such as vanillin, p-hydroxybenzoic acid, and other bio-based aromatic chemicals (Li Dan et al. 2021). In the food industry, ferulic acid is utilized as a natural antioxidant and preservative to improve food quality (de Oliveira et al. 2015). In the pharmaceutical industry, it is used for the synthesis of bioactive compounds with potential therapeutic properties (Dong and Huang 2022; Li Dan et al. 2021, Raj and Singh 2022; Zhai et al. 2023). Moreover, ferulic acid is gaining recognition as a renewable feedstock for biofuel production, as it can be converted into biofuels and other bio-based chemicals through microbial fermentation processes (Zheng et al. 2024). These applications make ferulic acid as a crucial raw material for sustainable chemical production, driving the development of eco-friendly alternatives in various industries. Despite their important roles of FEs, the discovery of promiscuous FEs, capable of acting on a wide range of substrates, such as 4-nitrophenyl ferulate, coniferyl p-coumarate, and 4-nitrophenyl feruloyl-L-arabinofuranoside, remains limited. This scarcity poses a significant challenge for biocatalysis and synthetic biology applications, hindering the development of efficient enzymatic processes for biomass conversion and valuable products synthesis (de Oliveira et al. 2015; Shukla et al. 2022). Addressing this challenge requires innovative approaches to enzyme discovery and design (Zhang Peng et al. 2023, Zhang Peng et al. 2020), as well as a deeper understanding of enzyme structure-function relationships. By leveraging enzyme similarity network analysis, molecular dynamics simulations and deep learning models, researchers can accelerate the identification of novel enzymes and optimize their catalytic activities for specific industrial applications (Wittmund et al. 2022). This concerted effort holds the promise of unlocking new pathways for sustainable biomanufacturing and advancing the bioeconomy.

In recent years, computational methods such as sequence-based prediction, MD simulations and deep learning strategy have gained prominence in enzyme specificity studies (Ebert and Pelletier 2017). For example, Li et al. employed large-scale sequence alignments between data from the Earth Microbiome Project and sequenced prokaryotic genomes to analyze the distribution, abundance, and diversity of genes encoding cellulases, xylanases, and chitinases in global prokaryotic communities (Li Dan-dan et al. 2023). Zhang et al. applied a comparative molecular dynamics approach to engineer glycosyltransferases, identifying key residue substitutions that expand substrate scope and enhance catalytic efficiency, enabling precise synthesis of valuable glycosides (Zhang Peng et al. 2024). However, limited efforts have been made to systematically explore substrate specificity in feruloyl esterases, highlighting the necessity of developing a computational framework specifically designed for feruloyl esterases, which integrates sequence analysis, MD simulations, and deep learning approaches to improve substrate specificity predictions and uncover potential applications in biofuel production and biomass valorization.

Enzyme similarity networks and phylogenetic analysis play crucial roles in enzyme discovery by providing insights into the evolutionary relationships and functional diversity of enzymes (Akiva et al. 2017; Zhang Li-Juan et al. 2022a). Enzyme similarity networks analyze the sequence and structural similarities among enzymes, facilitating the identification of enzyme families and functional clusters (Ashok et al. 2024; Mathieu et al. 2020). Phylogenetic trees depict the evolutionary history of enzymes to infer the relationships between different enzyme sequences and predict their functional properties (Kerk et al. 2021; Li X. X. et al. 2022). By utilizing enzyme similarity networks and phylogenetic trees, researchers can categorize enzymes into distinct groups based on their sequence, structure, and function. This classification provides a framework for exploring the biochemical diversity of enzymes and identifying potential candidates with novel catalytic activities (Kerk et al. 2021). Moreover, comparative analysis of enzyme sequences and structures can facilitate the explorations of the molecular basis of enzyme function and substrate specificity, guiding enzyme engineering and optimization efforts (Korany et al. 2020; Shi et al. 2023).

Molecular dynamics (MD) simulations offer a powerful tool for investigating the dynamic behaviors of enzymes at the atomic level. By simulating the motions and interactions of atoms within an enzyme structure over time, researchers can elucidate the conformational changes and dynamic fluctuations that govern enzyme function and substrate binding (Bhattacharjee et al. 2023; Jerves et al. 2021; Wang et al. 2024; Zhang Peng et al. 2022b). Molecular dynamics simulations provide valuable insights into enzyme-substrate interactions, catalytic mechanisms, and protein stability, aiding in the rational design of enzymes with improved properties (Cao et al. 2023; Li Jiao et al. 2020). Furthermore, molecular dynamics simulations can complement experimental studies by providing detailed atomic-level information that is often challenging to obtain experimentally. MD simulations can gain a comprehensive understanding of enzyme structure-function relationships and accelerate the discovery and optimization of enzymes for various biotechnological applications (Gilbert et al. 2022; Zaboli et al. 2021).

Deep learning, a subset of machine learning algorithms inspired by the structure and function of the human brain, has emerged as a powerful tool for enzyme discovery and design (Dallago and Yang 2023; Memon et al. 2020). Deep learning models, particularly neural networks, can analyze large-scale datasets of enzyme sequences, structures, and biochemical properties to predict enzyme functions, substrate specificities, and catalytic activities (Meng et al. 2023). By learning from complex patterns and relationships within the data, deep learning models can accurately classify enzymes, identify sequence-structure-function relationships, and prioritize candidate enzymes for experimental validation (Khan et al. 2021; Tao et al. 2020). Deep learning has revolutionized enzyme discovery by enabling rapid and cost-effective screening of enzyme libraries and protein sequence databases. Moreover, deep learning models can leverage transfer learning and ensemble methods to enhance prediction accuracy and generalize across different enzyme families and biochemical contexts (Shu et al. 2023). Harnessing the power of deep learning can accelerate the pace of enzyme discovery and design, facilitating the development of novel biocatalysts for diverse industrial applications (Han et al. 2022; Ming et al. 2023).

In this study, we explored 2085 FE sequences from the BRENDA database, a well-established repository that extensively documents experimentally validated enzymes. Many of these enzymes have been characterized through rigorous experimental methods, including kinetic assays and substrate specificity profiling, which provide a strong foundation for the computational analyses employed in this work. Beginning with enzymatic similarity network analysis, we identified three clusters. Notably, cluster 1 and cluster 3 showed significant sequence length differences. Phylogenetic analysis revealed a correlation between evolutionary classification and substrate spectrum. MD simulations highlighted structural variances between promiscuous and specific FEs. Leveraging deep learning models, we predicted 38 and 75 additional versatile FEs from cluster 1 and cluster 3 (probability score > 90%). Our findings emphasize the value of integrating phylogenetic, structural, and deep learning approaches for FEs mining, expanding our understanding of enzyme diversity and enhancing biocatalyst repertoire for synthetic applications.

## Analytical methods

### Enzyme similarity network analysis

2085 feruloyl esterases (FEs, EC 3.1.1.73) were retrieved from the BRENDA database (https://www.brenda-enzymes.org/) and the 2085 FE sequences were retrieved based on their functional annotation. To ensure the quality and representativeness of the dataset, sequences were filtered according to the following criteria: (1). Annotation quality of sequences was reviewed and confirmed; (2). The FE sequences shorter than 200 amino acids or longer than 800 amino acids were excluded, based on the main distribution range of the two FE classes; (3). Redundant sequences were removed by performing 100% sequence identity clustering using CD-HIT. The final selected sequences were initiated with enzyme similarity network analysis. The Enzyme Function Initiative-Enzyme Similarity Tool (EFI-EST) was then used to generate a sequence similarity network (SSN) for feruloyl esterases, and an all-by-all BLAST was performed to obtain the similarities between sequence pairs to calculate edge values to generate the SSN (Zallot et al. 2019). The resulting network was analyzed using Cytoscape 3.9.1, and SwissProt descriptions were used to organize the SSN and isolate a daughter cluster that contained the known feruloyl esterases. The default E-value was set to 5, and the filter value was set to 30.

### Phylogenetic analysis

Feruloyl esterases (FEs) from cluster 1 and cluster 3 were conducted multiple sequence alignments using MAFFT (https://mafft.cbrc.jp/alignment/server/) (Katoh et al. 2019). FastTree and Jukes-Cantor evolution model were used to construct maximum-likelihood phylogenetic trees (Price et al. 2009). To infer a tree for a protein alignment with the JTT + CAT model and to quickly estimate the reliability of each clade in the tree with the Shimodaira–Hasegawa test (Shimodaira and Hasegawa 1999). The resulting phylogenetic trees were visualized by iTOL (Letunic and Bork 2021).

### Computational analysis

AlphaFold2 server is used to generate the structures of feruloyl esterases (Jumper et al. 2021). The molecular dynamics (MD) simulations toward the characterized FEs were performed using the GROMACS 2020.6 software package (Justin 2018; Pronk et al. 2013). The protein was solvated using the SPC water model (Zielkiewicz 2005). The protein was centered in a 10 Å cubic box with periodic boundaries. The box was filled with around 30,394 water molecules. The system was neutralized by Na^+^ and Cl^−^ to achieve a net charge of zero. The AMBER14 force field was used for the residues of FEs (Maier et al. 2015). 5000 steps of the steepest descent followed by 5000 steps of conjugate gradients were used for energy minimization. The simulation of FEs was equilibrated by a 1 ns NVT ensemble followed by a 1 ns NPT ensemble, during which position restraints were applied to protein-heavy atoms. The production simulation was performed at 298 K for 100 ns with three replicates. The dominant conformations of FEs were obtained by clustering analysis of GROMACS with 0.1–0.25 nm cutoff. The dynamical cross-correlation matrix analysis was conducted by the previous work (Yu and Dalby 2018).

### Deep learning model

To predict the promiscuity of feruloyl esterases (FEs) and their catalytic substrate spectrum, we utilized the Substrate–Product Pair-based Enzyme Promiscuity Prediction (SPEPP) model. The model utilizes transfer learning and transformer architecture to analyze enzyme-substrate relationships. It was trained on a dataset comprising experimentally verified enzyme–substrate interactions from public databases, including BRENDA. The dataset was divided into training and validation sets, and cross-validation was employed to assess the model’s generalizability and prevent overfitting. Gradient-based optimization techniques were applied to fine-tune the model’s hyperparameters, with a loss function designed to balance prediction accuracy and interaction complexity. The model’s capabilities were integrated into the EnzyPick web server, providing an accessible tool for enzyme screening, particularly for users without programming expertise. EnzyPick is available at http://www.biosynther.com/enzypick/ (Xing et al. 2024). The three substrates: 4-nitrophenyl ferulate, coniferyl p-coumarate, and 4-nitrophenyl feruloyl-L-arabinofuranoside were selected as probes to test enzyme promiscuity. Enzymes with a probability score exceeding 90% for all three substrates were considered potentially promiscuous FEs.

## Results and discussion

### Enzyme similarity networks of feruloyl esterases

**Fig. 1:**
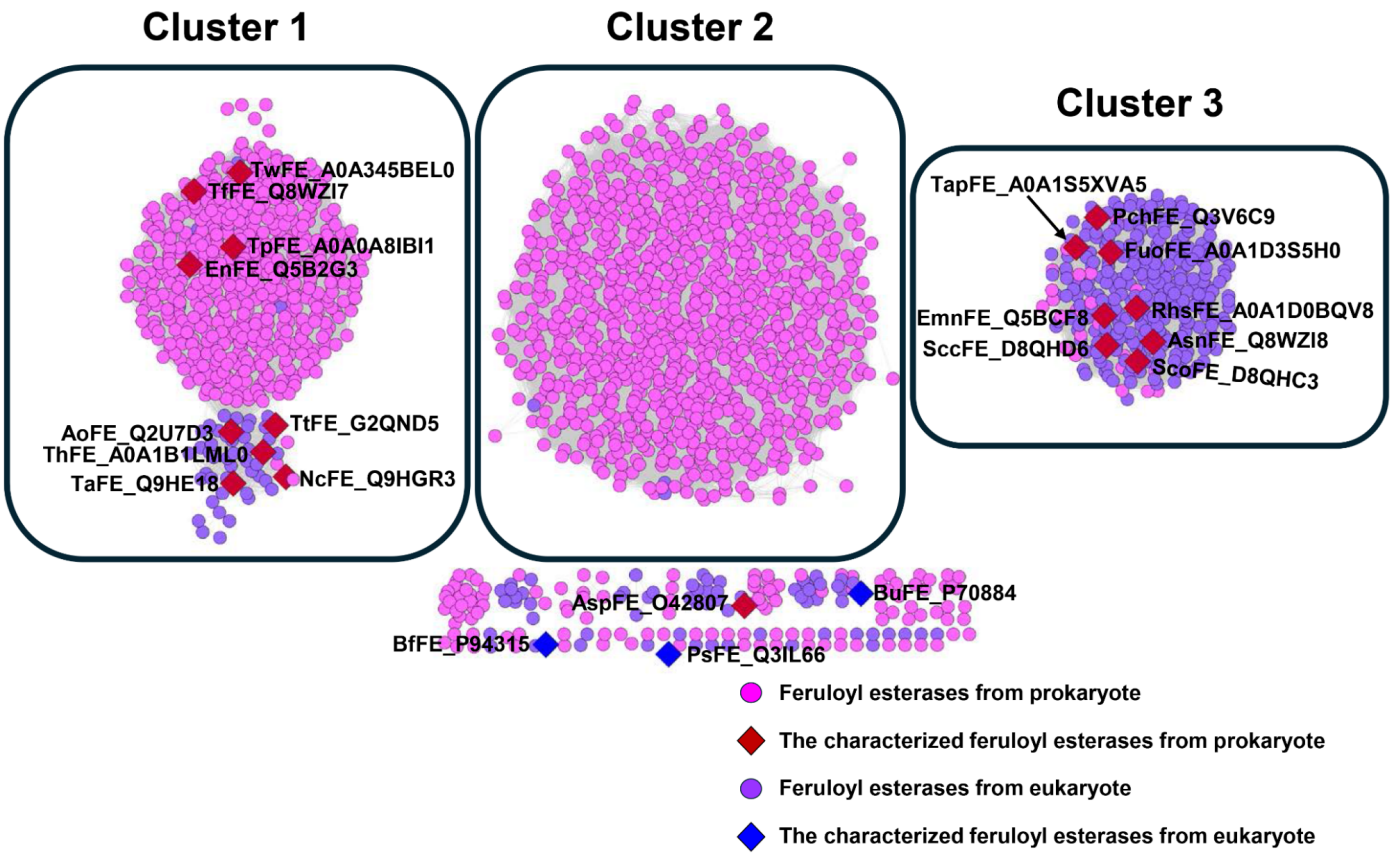
Enzyme similarity network analysis of feruloyl esterases (FEs, EC 3.1.1.73). FEs from prokaryotes are labeled pink and FEs from eukaryotes are labeled blue. The characterized FEs from prokaryotes are labeled red (diamond) and the characterized FEs from eukaryotes are labeled blue (diamond). The UniProt IDs of the characterized FEs are also labeled

To explore the general characteristics of feruloyl esterases (FEs), we initially obtained all 2085 FE sequences (EC 3.1.1.73) from BRENDA database. The enzyme similarity network showed that the FE sequences were divided three main clusters (Fig. 1). FEs from cluster 1 and cluster 2 were predominantly derived from prokaryotes, while a subcluster (a division of main cluster) from cluster 1 was mainly from eukaryotes. Notably, the FEs from cluster 3 were primarily of eukaryotic origin. Through retrieving characterized FEs from the BRENDA database (EC 3.1.1.73), a total of 21 characterized FEs were identified for further analysis, with 17 representative enzymes distributed across clusters. Specifically, 9 FEs were identified in cluster 1, and 8 enzymes were found in cluster 3. Surprisingly, no characterized enzymes were distributed in cluster 2. Among all cluster 1 entities, 5 FEs (AoFE, ThFE, TaFE, TtFE and NcFE) were distributed within eukaryotic subcluster, while 4 enzymes (TwFE, TfFE, TpFE and EnFE) were distributed within prokaryotic subcluster. Interestingly, all 9 enzymes in cluster 1 were derived from prokaryotes, although they belonged to either the prokaryote subcluster or the eukaryote subcluster. Similarly, cluster 3, which represented a typical eukaryotic cluster, comprised 8 characterized enzymes (TapFE, PchFE, FuoFE, EmnFE, SccFE, RhsFE, AsnFE and ScoFE) exclusively sourced from prokaryotes. We hypothesize that there is horizontal gene transfer between prokaryotic and eukaryotic clusters. Intriguingly, upon closer examination of cluster 1 and cluster 3 using characterized representative enzymes, we observed significant differences in sequence length. FE sequences in cluster 1 were approximately 300 amino acids in length, whereas those in cluster 3 exceeded 500 amino acids. This implies that the functional entities within cluster 1 or 3 may represent two distinct classes of FEs, each possessing unique functional characteristics.

### Phylogenetic classification of the characterized feruloyl esterases with different substrate scope

**Fig. 2:**
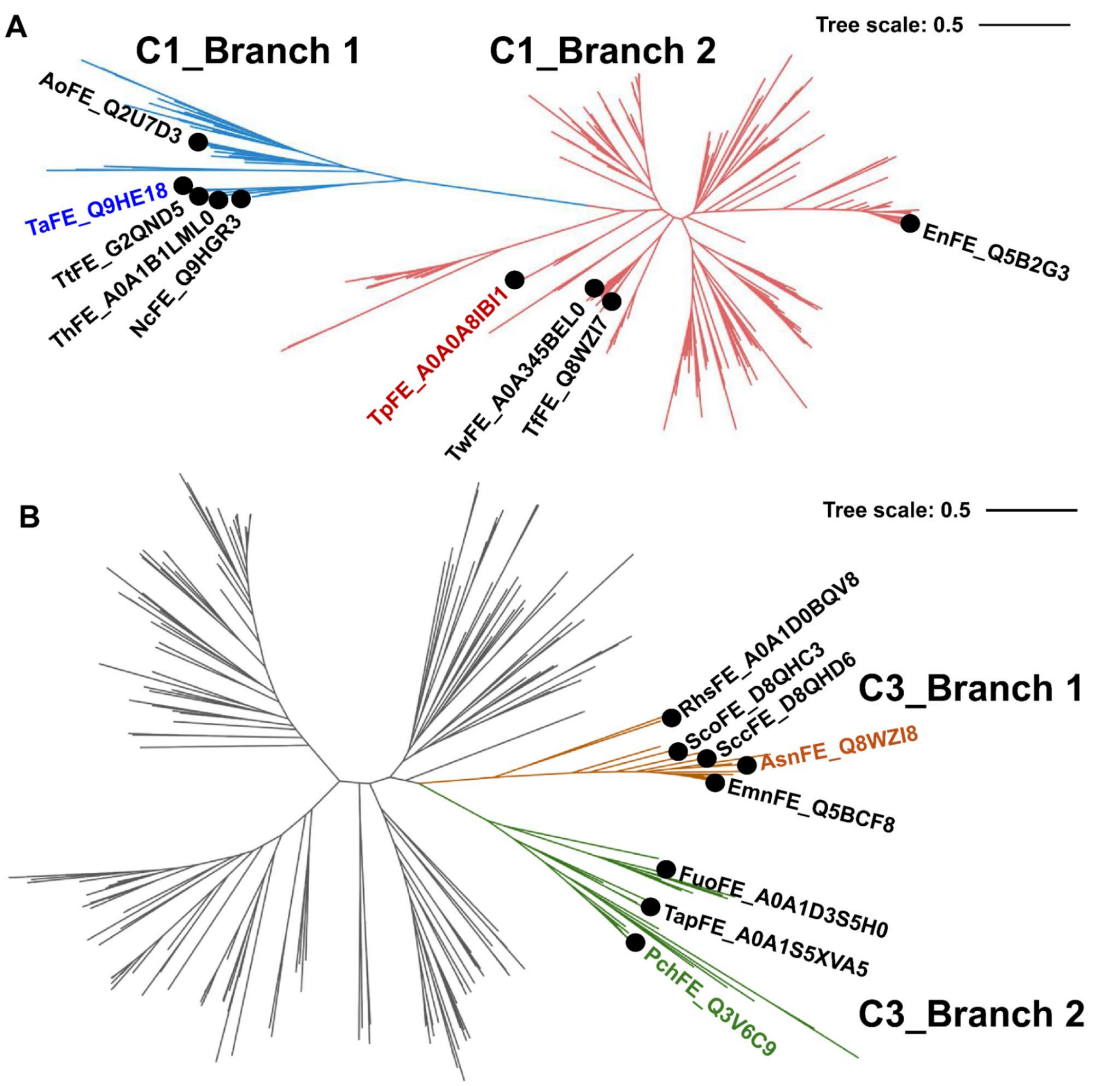
Phylogenetic tree analysis of feruloyl esterases from cluster 1 and cluster 3. (**A**) The phylogenetic tree of feruloyl esterases from cluster 1, the characterized enzymes with different substrate scope are labeled in C1_Branch 1 (blue) and C1_Branch 2 (red). (**B**) The phylogenetic tree of feruloyl esterases from cluster 3, the characterized enzymes with different substrate scope are also labeled in C3_Branch 1 (yellow) and C3_Branch 2 (green)

To further explore the structural and functional characteristics of FEs in clusters 1 and 3, we constructed phylogenetic trees for these two clustered FEs (Fig. 2). Like the enzyme similarity network, we observed that the phylogenetic tree of cluster 1 were also divided into two subbranches, C1_Branch 1 and C1_Branch 2. 5 representative FEs (AoFE, ThFE, TaFE, TtFE, and NcFE) were situated within the subbranch C1_Branch 1, while 4 representative enzymes (TwFE, TfFE, TpFE, and EnFE) were located within the subbranch C1_Branch 2 (Fig. 2A). In contrast, all 8 FEs in cluster 3 were concentrated within two subbranches (C3_Branch 1 and C3_Branch 2) of the evolutionary tree (Fig. 2B). To delve deeper into the characteristics of enzymes across different branches, we gathered substrate catalytic features for all representative FEs. Surprisingly, the 5 FEs (AoFE, ThFE, TaFE, TtFE and NcFE) situated within the subbranch C1_Branch 1 exhibited a broad substrate scope in cluster 1, whereas the 4 FEs (TwFE, TfFE, TpFE and EnFE) within the subbranch C1_Branch 2 displayed a narrow substrate spectrum. Similarly, the 5 FEs (EmnFE, SccFE, RhsFE, AsnFE and ScoFE) within subbranch C3_Branch 1 showcased a broader substrate spectrum compared to the 3 FEs (TapFE, PchFE and FuoFE) within subbranch C3_Branch 2 in cluster 3 (Table S1). This discovery highlights the significance of understanding evolutionary divergence’s influence on the functional diversity of FEs within clusters 1 and 3. Therefore, we selected the FEs with the broadest and narrowest substrate spectra in clusters 1 and 3 for further investigation (TaFE with the broadest substrate scope and TpFE with the narrowest substrate scope in cluster 1; AsnFE with the broadest substrate scope and PchFE with the narrowest substrate scope in cluster. Evolutionary divergence within these clusters of FEs is closely associated with substrate preference and functional adaptation. The FEs of broad substrate range in branches reflects adaptations to varying environmental pressures, while the FEs of narrow substrate specificity suggests more specialized roles related to the metabolic needs of their organisms.

### Structure-based for molecular dynamic characterization of feruloyl esterases with different substrate scope

**Fig. 3:**
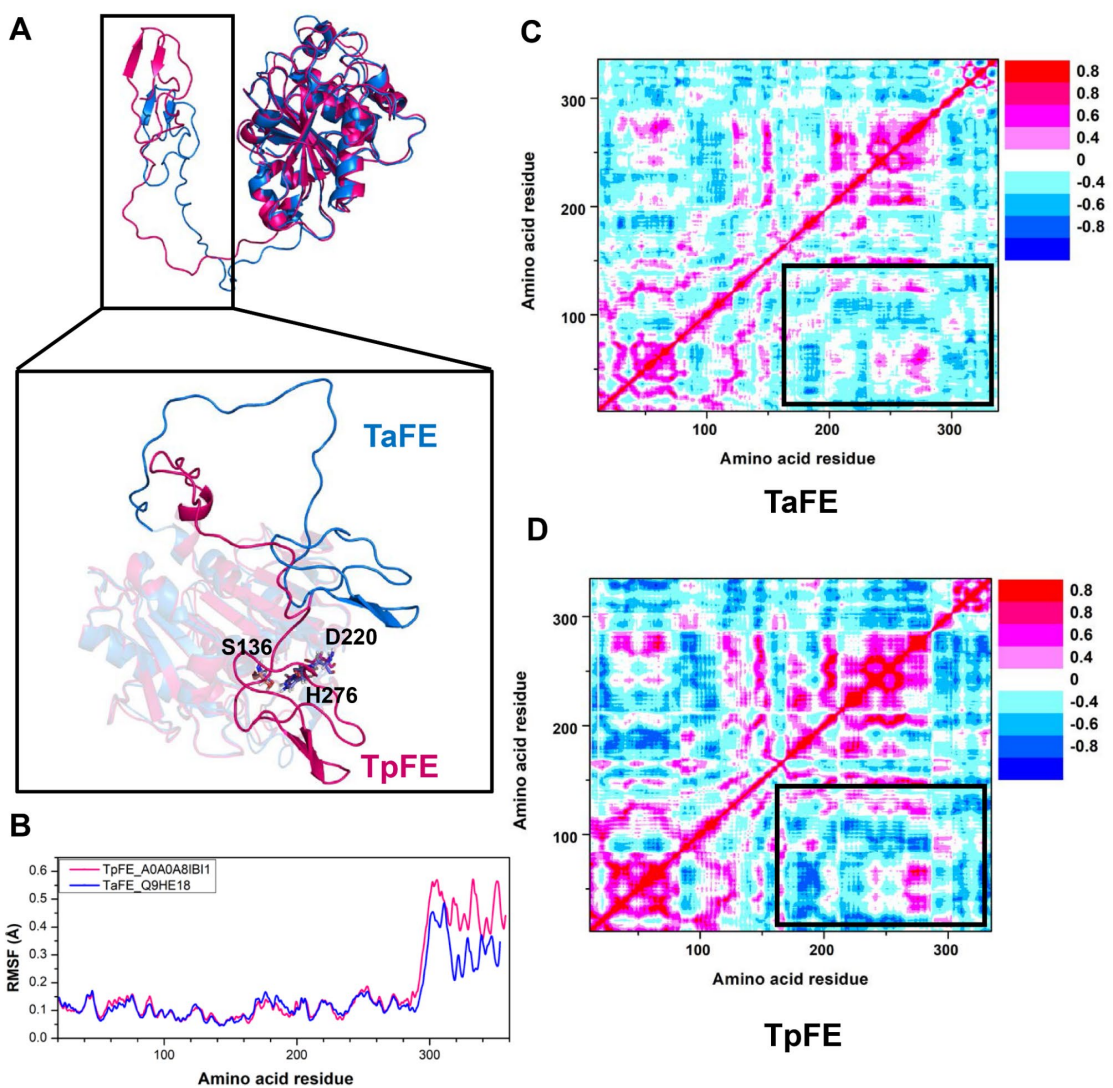
Molecular dynamics analysis of representative FEs (TpFE_A0A0A8IBI1 and TaFE_Q9HE18) with different substrate scope in cluster 1. **A**) The comparison of dominant conformations of TaFE (blue) and TpFE (red) with the conserved catalytic triad (Ser136, Asp220, and His276, two FEs are the same). **B**) The RMSF values for the α-carbon of each residue in TaFE (blue) and TpFE (red) after three parallel 100 ns MD simulations. **C**) and **D**) The dynamical cross-correlation matrix analysis of TaFE (**C**) and TpFE (**D**). The cross-correlation regions are labeled with rectangles

TaFE and TpFE are two FEs located in cluster 1 that exhibit evolutionary divergence in enzyme similarity networks and phylogenetic trees. While TaFE exhibited a broad substrate spectrum, TpFE showed substrate specificity. To explore the correlation between the structures of these two enzymes and their catalytic features, we utilized AlphaFold2 to perform protein modeling for TaFE and TpFE, followed by structural alignment (Fig. 3A). We observed that both TaFE and TpFE exhibit a loose loop region and a terminal α-helix at the end of their structures, forming a “lid” structure (long loop that differ from the main structure of the protein). Structural comparison of TaFE and TpFE revealed that the lid structure in TpFE partially obstructs access to the conserved catalytic triad (Ser136, Asp220, and His276), which may contribute to its narrower substrate spectrum compared to TaFE. To further understand the dynamic information of residues of these two FEs, we conducted 100 ns molecular dynamics simulations for TaFE and TpFE, and then characterized the dynamic behaviors of each amino acids using root mean square fluctuation (RMSF) values (Fig. S1). We found that both TaFE and TpFE show similar RMSF values for the first 300 amino acids, indicating consistent flexibility of these amino acids. However, significant differences in flexibility were observed in the loop region and terminal α-helix after 300 amino acids, with TaFE exhibiting lower flexibility compared to TpFE (Fig. 3B). Hence, high flexibility and the positioning of the “lid” structure can lead to unstable protein conformation and steric hindrance to substrate access, collectively reducing substrate promiscuity. As the “lid” structure is located at the terminal end of the entire protein, we further explored the long-range correlation between the “lid” structure and the main body structures of both TaFE and TpFE. We separately calculated the dynamical cross-correlation matrices of the two FEs. Interestingly, the amino acid correlation of TaFE is much lower than that of TpFE (Fig. 3C and D). The strong correlation (positive or negative) between the “lid” structure and the main body structure, may also result in poor substrate promiscuity. Overall, the high flexibility, positioning of the lid structure, and its correlation with the main body are critical for the substrate promiscuity of FEs.

**Fig. 4:**
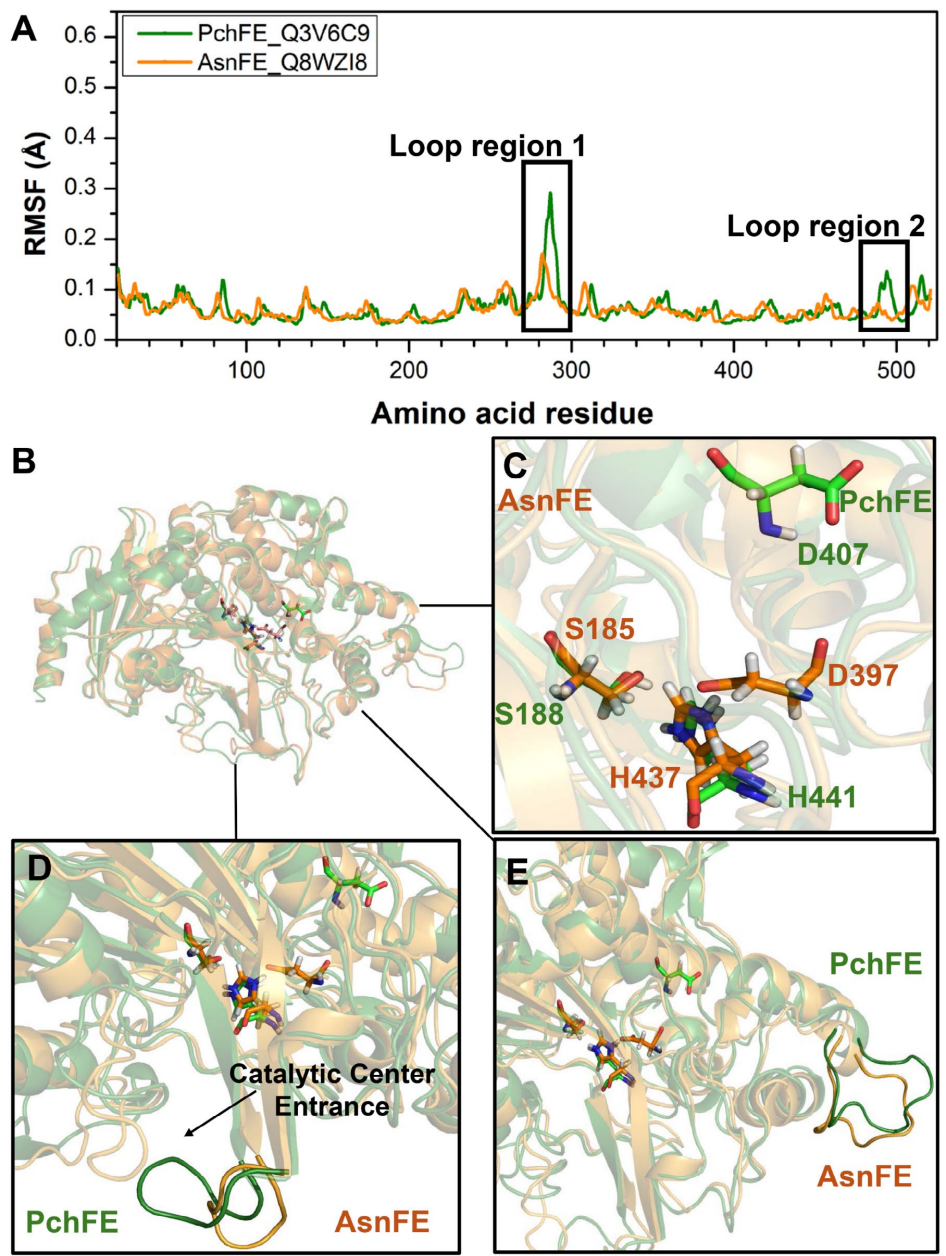
Molecular dynamics analysis of representative FEs with different substrate scope in cluster 3. (**A**) The RMSF values for the α-carbon of each residue in PchFE (green) and AsnFE (orange) after three parallel 100 ns MD simulations. (**B**) The structural alignment of dominant conformations of PchFE (green) and AsnFE (orange). The triplet catalytic residues Ser-Asp-His are labeled. (**C**) Positional relationships of the triplet catalytic residues Ser-Asp-His in PchFE (green) and AsnFE (orange). (**D**) Differences in the position of loop region 1 towards the catalytic center for PchFE (green) and AsnFE (orange). (**E**) Comparison of the relative positions of loop region 2 in PchFE (green) and AsnFE (orange)

To further investigate the differential characteristics of FEs in cluster 3, we analyzed two representative FEs, AsnFE and PchFE, as they exhibit different substrate scope. AsnFE exhibited a broad substrate spectrum, while PchFE showed a narrow substrate spectrum. Similarly, we employed AlphaFold2 for protein modeling of AsnFE and PchFE, followed by 100 ns molecular dynamics simulations. By comparing RMSF values (Fig. S2), we observed significant differences in flexibility in two loop regions, namely Loop region 1 and Loop region 2 of AsnFE and PchFE (Fig. 4A). In both regions, AsnFE exhibited lower flexibility compared to PchFE, which is consistent with the results observed in FEs from cluster 1, the FEs with broader substrate spectrum usually showed lower loop flexibility (Figs. 3B and 4A).

Through molecular dynamics simulations, we further obtained the dominant conformations of AsnFE and PchFE and compared their structural differences (Fig. 4B). By analyzing the triplet catalytic residues Ser-Asp-His, we found a higher degree of overlap between serines (S185 from AsnFE and S188 from PchFE) and histidines (H437 from AsnFE and H441 from PchFE). The catalytic residue aspartate (D397) was closer to S185 and H437 in AsnFE, whereas the catalytic residue aspartate (D407) was far from S188 and H441 in PchFE (Fig. 4C). Additionally, Loop region 1 of PchFE is positioned closer to the entrance of the catalytic center compared to that of AsnFE, which may hinder substrate entry into the catalytic center, resulting in a narrower substrate spectrum for PchFE (Fig. 4D). Since Loop region 2 of both AsnFE and PchFE are distanced from the catalytic center, Loop region 2 did not exhibit significant differences (Fig. 4E).

**Fig. 5:**
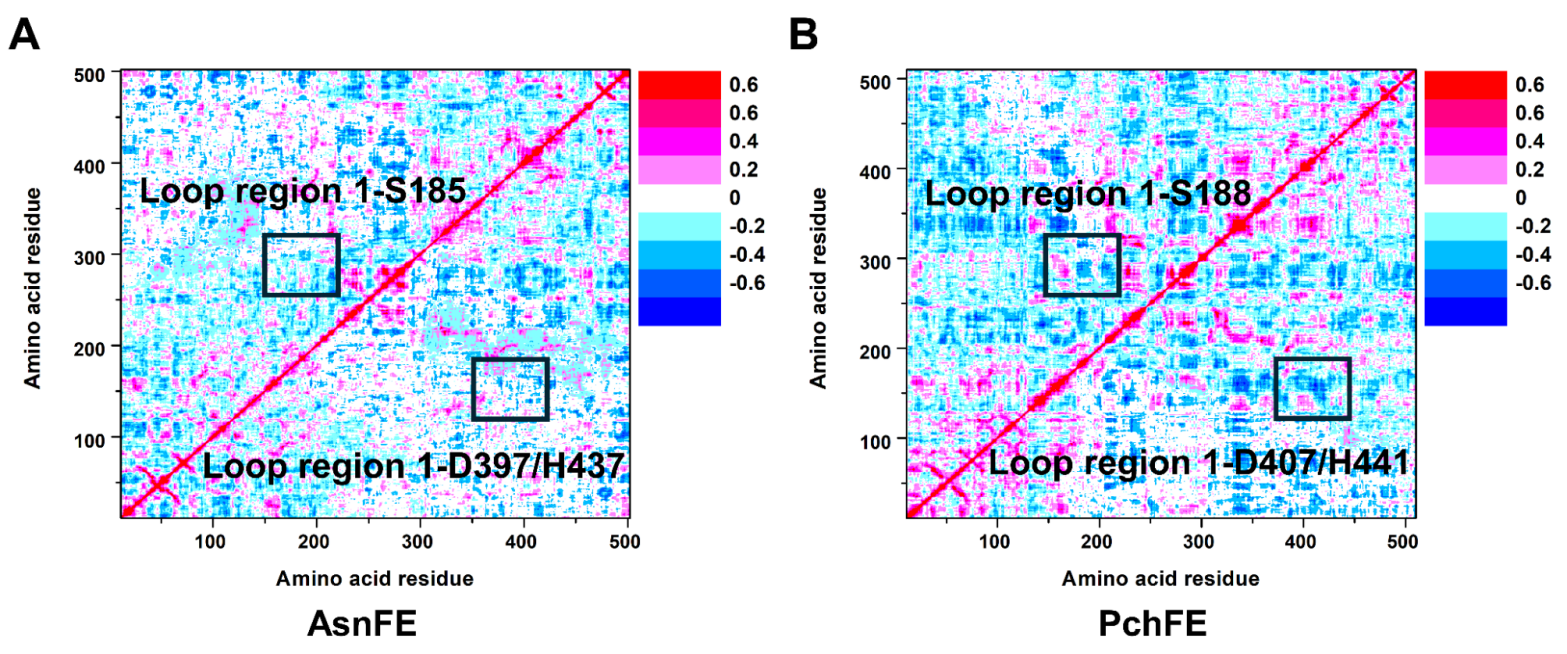
The dynamical cross-correlation matrix analysis of AsnFE (**A**) and PchFE (**B**). The cross-correlation regions are labeled with rectangles

We further investigated the dynamic long-range correlation between loop regions (loop region 1 and loop region 2) and the catalytic center in both AsnFE and PchFE. We separately calculated the dynamical cross-correlation matrices of these two FEs. We found that, in both AsnFE and PchFE, Loop region 2 did not exhibit significant correlation with the three catalytic residue regions. However, notably, loop region 1 of PchFE showed strong negative distant correlation with the catalytic residues serine (S188), aspartate (D407), and histidine (H441). In contrast, this distant correlation was less pronounced in AsnFE (Fig. 5A and B). Therefore, the high flexibility of the loop regions may propagate to the catalytic center, resulting in an unstable protein conformation for FEs and poor substrate promiscuity. Conversely, the stability of loop structures and weak correlation with catalytically relevant residues may enhance the substrate promiscuity of FEs, expanding their substrate scope.

### Mining of versatile feruloyl esterases using deep learning model

**Fig. 6:**
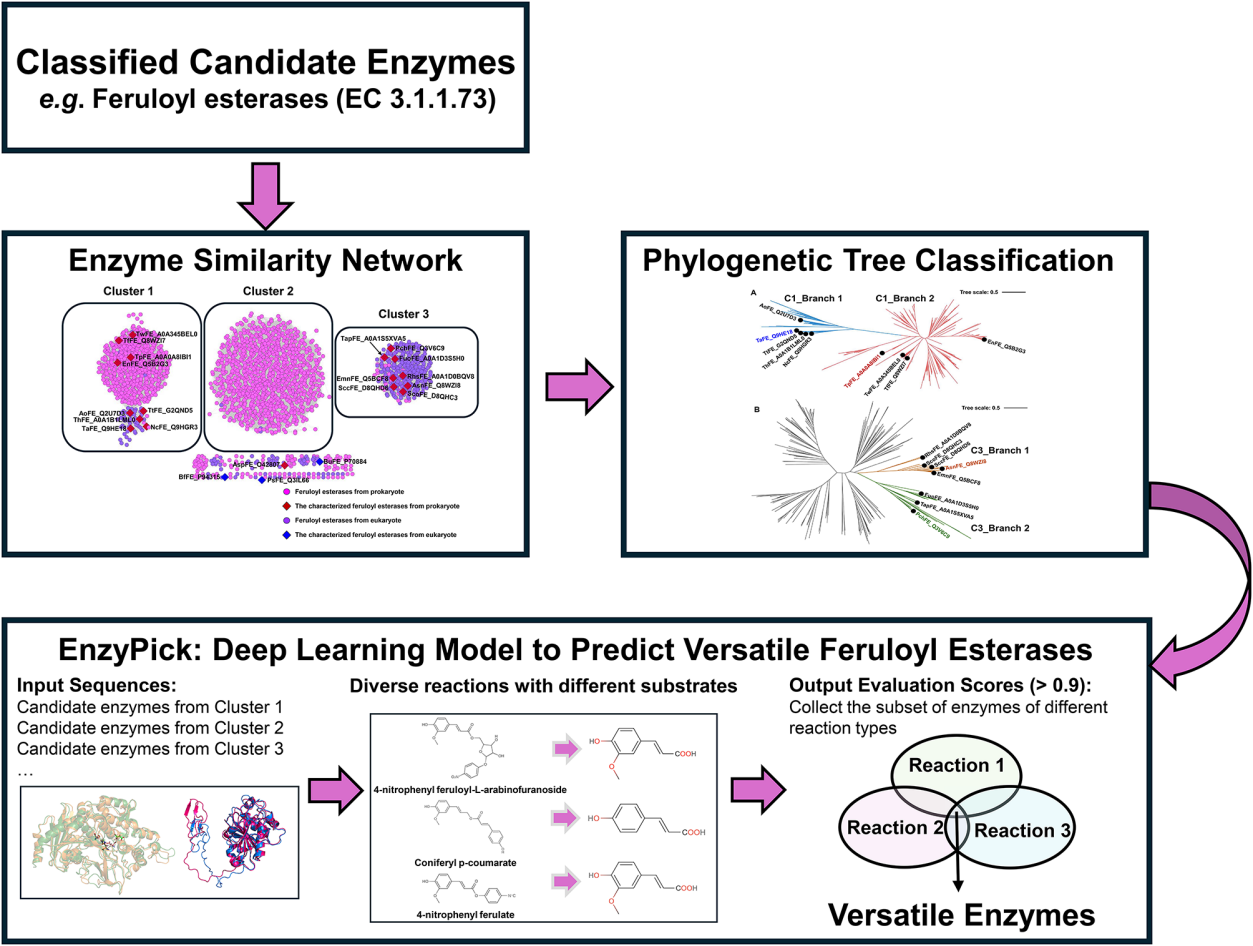
The workflow of the phylogenetic and structural feature-based deep learning strategy for mining of the versatile feruloyl esterases

The use of molecular modeling and dynamic simulations can predict whether FEs belong to versatile enzymes, but this method often cannot be applied on a large scale for the prediction of a vast number of FEs. Herein, we employed the deep learning model of EnzyPick (Enzyme Selection Tool (Xing et al. 2024) to predict potential versatile FEs (Fig. 6). Step 1, all FEs (3.1.1.73) were retrieved from the BRENDA database. Step 2, enzyme similarity network analysis was conducted to preliminarily cluster all FEs, and the structural or catalytic features of different clusters were determined using characterized FEs. Step 3, phylogenetic tree analysis of specific clusters was performed to locate FEs with different catalytic features, focusing on identifying FEs with broad substrate spectrum, particularly those exhibiting substrate promiscuity. Step 4, specific types of reactions were determined, and a deep learning model (such as EnzyPick) was applied to screen potential versatile FEs compatible with each reaction types. Here, we selected three reaction types, represented by the substrates 4-nitrophenyl ferulate, coniferyl p-coumarate, and 4-nitrophenyl feruloyl-L-arabinofuranoside, and predicted potential versatile FEs using the EnzyPick deep learning model. These substrates were chosen as probes to comprehensively test the enzyme promiscuity and substrate specificity of FEs, highlighting their versatility in catalyzing a wide range of ester bond hydrolysis reactions relevant to both industrial and environmental applications. The FEs with probability scores exceeding 90% in all three reaction types were selected, and 38 and 75 potential versatile FEs in clusters 1 and 3 were predicted, respectively (Table S2 and Table S3, highlighted in yellow).

## Conclusion

Feruloyl esterases (FEs, EC 3.1.1.73) play vital roles in biological synthesis and metabolism, yet identifying versatile FEs capable of catalyzing a wide range of substrates poses a challenge. This study integrated multiple methodologies to address this challenge effectively. Initially, an enzyme similarity network analysis revealed distinct clusters, with subsequent phylogenetic analysis linking phylogenetic classification with substrate promiscuity. Structural dynamics differences between promiscuous and substrate-specific FEs were explored using molecular dynamics simulations and dynamical cross-correlation matrix analysis. Additionally, the employment of deep learning models significantly expanded the repertoire of versatile FEs, identifying 38 and 75 potential candidates from cluster 1 and cluster 3, respectively, with high probability scores. The findings underscore the importance of integrating phylogenetic and structural features with deep learning approaches for mining versatile FEs, thereby shedding light on unexplored enzymatic diversity. This holistic approach not only enhances our understanding of enzyme functionality but also expands the toolbox of biocatalysts for diverse practical applications. For instance, the identification of FEs with broad substrate specificity can drive advancements in biomass degradation for biofuel production, enabling more efficient conversion of lignocellulosic materials into bioethanol or biodiesel. Additionally, these versatile enzymes can be applied in the synthesis of value-added chemicals, such as phenolic compounds and antioxidants, which are widely used in the pharmaceutical, cosmetic, and food industries. These applications not only showcase the industrial relevance of FEs but also highlight their potential in reducing environmental impact and advancing green chemistry principles across a range of biotechnological and bioengineering processes.

## Electronic supplementary material

Below is the link to the electronic supplementary material.

Supplementary Material 1

Supplementary Material 2

Supplementary Material 3

Supplementary Material 4

Supplementary Material 5

## Data Availability

The datasets generated and analysed during the current study are available in the BRENDA repository (https://www.brenda-enzymes.org/) and CAZy repository (http://www.cazy.org/).

## Abbreviations

FEs: Feruloyl esterases
MD: Molecular dynamics
iTOL: Interactive Tree Of Life
SPC: Simple Point Charge
NVT: Constant Number of particles, Volume, and Temperature
NPT: Constant Number of particles, Volume, and Temperature
