# Supplementary material for: Mining versatile feruloyl esterases: phylogenetic classification, structural features, and deep learning model
Source: Bioresour Bioprocess. 2025 Jan 29;12(1):7. doi: 10.1186/s40643-024-00835-8 (PMC11780051; doi:10.1186/s40643-024-00835-8)
Supplement: Supplementary file 1 — Supplementary Material 1 [file 40643_2024_835_MOESM1_ESM.pdf]

# ~~Phylogenetic and Structural Feature-based Deep Learning Guided Mining of Versatile Feruloyl Esterases~~Mining Versatile Feruloyl Esterases: Phylogenetic Classification, Structural Features, and Deep Learning Model

Liang Guo[1], Yuxin Dong[2], Deyong Zhang[1], Xinrong Pan[1], Xinjie Jin[3], Xinyu Yan[4], Yin Lu[1]*

1. Key Laboratory of Pollution Exposure and Health Intervention of Zhejiang Province, College of Biological and Environment Engineering, Zhejiang Shuren University, Hangzhou 310015, China

2. Jinan No.1 High School, Jinan 250014, China

3. College of Life and Environmental Science, Wenzhou University, Wenzhou 325035, Zhejiang, China

4. College of Agriculture, Yangtze University, Jingzhou 434000, Hubei Province, China

*Corresponding authors

Yin Lu (luyin@zjsru.edu.cn) ~~(luyin@zjsru.edu.cn)(luyin@zjsru.edu.cn)(luyin@zjsru.edu.cn)~~

## Abstract

Feruloyl esterases (FEs, EC 3.1.1.73) play a crucial role in biological synthesis and metabolism. However, the identification of versatile FEs, capable of catalyzing a wide range of substrates, remains a challenge. In this study, we obtained 2085 FE sequences from the BRENDA database and initiated with an enzyme similarity network analysis, revealing three main clusters (1-3). Notably, both cluster 1 and cluster 3 included the characterized FEs~~representatives~~, which exhibited significant differences in sequence length. Subsequent phylogenetic analysis of these clusters unveiled a correlation between phylogenetic classification and substrate promiscuity, and enzymes with broad substrate scope tended to ~~cluster~~ locate within specific branches of the phylogenetic tree. Further, molecular dynamics simulations and dynamical cross-correlation matrix analysis were employed to explore structural dynamics differences between promiscuous and substrate-specific FEs. Finally, to expand the repertoire of versatile FEs, we employed deep learning models to predict potentially promiscuous enzymes and identified 38 and 75 potential versatile FEs from cluster 1 and cluster 3 with a probability score exceeding 90%. Our findings underscore the utility of integrating phylogenetic and structural features with deep learning approaches for mining versatile FEs, shedding light on unexplored enzymatic diversity and expanding the repertoire of biocatalysts for synthetic applications.

**Key words**: Feruloyl esterases, Enzyme similarity network, Phylogenetic analysis, Molecular dynamics simulations, Deep learning

## 1. Introduction

Feruloyl esterases (FEs, EC 3.1.1.73) are pivotal enzymes with significant implications in various biological processes(Oliveira et al. 2019). These enzymes catalyze the hydrolysis of ester bonds between hydroxycinnamic acids and plant cell wall polysaccharides, contributing to the degradation of lignocellulosic biomass and the release of ferulic acid(Gopalan et al. 2015). Ferulic acid ~~(de Oliveira et al. 2015)~~is a key aromatic compound widely used as a precursor in the synthesis of various valuable products, such as vanillin, p-hydroxybenzoic acid, and other bio-based aromatic chemicals(Li Dan et al. 2021). In the food industry, ferulic acid is utilized as a natural antioxidant and preservative to improve food quality(de Oliveira et al. 2015). In the pharmaceutical industry, it is used for the synthesis of bioactive compounds with potential therapeutic properties(Dong and Huang 2022, Li Dan et al. 2021, Raj and Singh 2022, Zhai et al. 2023). Moreover, ferulic acid is gaining recognition as a renewable feedstock for biofuel production, as it can be converted into biofuels and other bio-based chemicals through microbial fermentation processes(Zheng et al. 2024). These applications make ferulic acid as a crucial raw material for sustainable chemical production, driving the development of eco-friendly alternatives in various industries.~~Ferulic acid serves as a precursor for the synthesis of valuable compounds, including vanillin and other aromatic chemicals, making FEs crucial for biotechnological applications such as biofuel production, pharmaceutical synthesis, and food processing(Adilah et al. 2022, Dong and Huang 2022, Zhai et al. 2023).~~ Despite their important roles of FEs, the discovery of promiscuous ~~versatile~~ FEs, capable of acting on a wide range of substrates, such as 4-nitrophenyl ferulate, coniferyl p-coumarate, and 4-nitrophenyl feruloyl-L-arabinofuranoside, remains limited. This scarcity poses a significant challenge for biocatalysis and synthetic biology applications, hindering the development of efficient enzymatic processes for biomass conversion and valuable products synthesis(de

Oliveira et al. 2015, Shukla et al. 2022). Addressing this challenge requires innovative approaches to enzyme discovery and design(Zhang Peng et al. 2023, Zhang Peng et al. 2020), as well as a deeper understanding of enzyme structure-function relationships. By leveraging enzyme similarity network analysis, molecular dynamics simulations ~~advanced computational techniques~~ and deep learning models, researchers can accelerate the identification of novel enzymes and optimize their catalytic activities for specific industrial applications(Wittmund et al. 2022). This concerted effort holds the promise of unlocking new pathways for sustainable biomanufacturing and advancing the bioeconomy.

In recent years, computational methods such as sequence-based prediction, MD simulations and deep learning strategy have gained prominence in enzyme specificity studies(Ebert and Pelletier 2017). For example, ~~(Chen et al. 2019)~~Li *et al.* employed large-scale sequence alignments between data from the Earth Microbiome Project and sequenced prokaryotic genomes to analyze the distribution, abundance, and diversity of genes encoding cellulases, xylanases, and chitinases in global prokaryotic communities(Li Dan-dan et al. 2023). Zhang *et al.* applied a comparative molecular dynamics approach to engineer glycosyltransferases, identifying key residue substitutions that expand substrate scope and enhance catalytic efficiency, enabling precise synthesis of valuable glycosides(Zhang Peng et al. 2024). However, limited efforts have been made to systematically explore substrate specificity in feruloyl esterases, highlighting the necessity of developing a computational framework specifically designed for feruloyl esterases, which integrates sequence analysis, MD simulations, and deep learning approaches to improve substrate specificity predictions and uncover potential applications in biofuel production and biomass valorization.

Enzyme similarity networks and phylogenetic analysis play crucial roles in enzyme discovery by providing insights into the evolutionary relationships and functional diversity of enzymes(Akiva et al. 2017, Zhang Li-Juan et al. 2022a). Enzyme similarity networks analyze the sequence and structural similarities among enzymes, facilitating the identification of

enzyme families and functional clusters(Ashok et al. 2024, Mathieu et al. 2020). Phylogenetic trees depict the evolutionary history of enzymes to infer the relationships between different enzyme sequences and predict their functional properties(Kerk et al. 2021, Li X. X. et al. 2022). By utilizing enzyme similarity networks and phylogenetic trees, researchers can categorize enzymes into distinct groups based on their sequence, structure, and function. This classification provides a framework for exploring the biochemical diversity of enzymes and identifying potential candidates with novel catalytic activities(Kerk et al. 2021). Moreover, comparative analysis of enzyme sequences and structures can facilitate the explorations of the molecular basis of enzyme function and substrate specificity, guiding enzyme engineering and optimization efforts(Korany et al. 2020, Shi et al. 2023).

Molecular dynamics (MD) simulations offer a powerful tool for investigating the dynamic behaviors of enzymes at the atomic level. By simulating the motions and interactions of atoms within an enzyme structure over time, researchers can elucidate the conformational changes and dynamic fluctuations that govern enzyme function and substrate binding(Bhattacharjee et al. 2023, Jerves et al. 2021, Wang et al. 2024, Zhang Peng et al. 2022b). Molecular dynamics simulations provide valuable insights into enzyme-substrate interactions, catalytic mechanisms, and protein stability, aiding in the rational design of enzymes with improved properties(Cao et al. 2023, Li Jiao et al. 2020). Furthermore, molecular dynamics simulations can complement experimental studies by providing detailed atomic-level information that is often challenging to obtain experimentally. MD simulations can gain a comprehensive understanding of enzyme structure-function relationships and accelerate the discovery and optimization of enzymes for various biotechnological applications(Gilbert et al. 2022, Zaboli et al. 2021).

Deep learning, a subset of machine learning algorithms inspired by the structure and function of the human brain, has emerged as a powerful tool for enzyme discovery and design(Dallago and Yang 2023, Memon et al. 2020). Deep learning models, particularly neural networks, can analyze large-scale datasets of enzyme sequences, structures, and biochemical properties to

predict enzyme functions, substrate specificities, and catalytic activities(Meng et al. 2023). By learning from complex patterns and relationships within the data, deep learning models can accurately classify enzymes, identify sequence-structure-function relationships, and prioritize candidate enzymes for experimental validation(Khan et al. 2021, Tao et al. 2020). Deep learning has revolutionized enzyme discovery by enabling rapid and cost-effective screening of enzyme libraries and protein sequence databases. Moreover, deep learning models can leverage transfer learning and ensemble methods to enhance prediction accuracy and generalize across different enzyme families and biochemical contexts(Shu et al. 2023). Harnessing the power of deep learning can accelerate the pace of enzyme discovery and design, facilitating the development of novel biocatalysts for diverse industrial applications(Han et al. 2022, Ming et al. 2023).

In this study, we explored 2085 FE sequences from the BRENDA database, a well-established repository that extensively documents experimentally validated enzymes. Many of these enzymes have been characterized through rigorous experimental methods, including kinetic assays and substrate specificity profiling, which provide a strong foundation for the computational analyses employed in this work. Beginning with enzymatic similarity network analysis, we identified three clusters. Notably, cluster 1 and cluster 3 showed significant sequence length differences. Phylogenetic analysis revealed a correlation between evolutionary classification and substrate spectrum~~versatility~~. MD simulations highlighted structural variances between promiscuous and specific FEs. Leveraging deep learning models, we predicted 38 and 75 additional versatile FEs from cluster 1 and cluster 3 (probability score >90%). Our findings emphasize the value of integrating phylogenetic, structural, and deep learning approaches for FEs mining, expanding our understanding of enzyme diversity and enhancing biocatalyst repertoire for synthetic applications.

## 2. Analytical M~~m~~ethods

### 2.1. Enzyme similarity network analysis

2085 feruloyl esterases (FEs, EC 3.1.1.73) were retrieved from the BRENDA database (https://www.brenda-enzymes.org/) and the 2085 FE sequences were retrieved based on their functional annotation. To ensure the quality and representativeness of the dataset, sequences were filtered according to the following criteria: (1). Annotation quality of sequences was reviewed and confirmed; (2). The FE sequences shorter than 200 amino acids or longer than 800 amino acids were excluded, based on the main distribution range of the two FE classes; (3). Redundant sequences were removed by performing 100% sequence identity clustering using CD-HIT. The final selected sequences were initiated with enzyme similarity network analysis~~initiated with an enzyme similarity network analysis (Zallot et al. 2019)~~. The Enzyme Function Initiative-Enzyme Similarity Tool (EFI-EST) was then used to generate a sequence similarity network (SSN) for feruloyl esterases, and an all-by-all BLAST was performed to obtain the similarities between sequence pairs to calculate edge values to generate the SSN(Zallot et al. 2019). The resulting network was analyzed using Cytoscape 3.9.1, and SwissProt descriptions were used to organize the SSN and isolate a daughter cluster that contained the known feruloyl esterases. The default E-value was set to 5, and the filter value was set to 30.

### 2.2. Phylogenetic analysis

Feruloyl esterases (FEs) from cluster 1 and cluster 3 were conducted multiple sequence alignments using MAFFT (https://mafft.cbrc.jp/alignment/server/)(Katoh et al. 2019). FastTree and Jukes-Cantor evolution model were used to construct maximum-likelihood phylogenetic trees(Price et al. 2009). To infer a tree for a protein alignment with the JTT+CAT model and to quickly estimate the reliability of each clade in the tree with the Shimodaira–Hasegawa test(Shimodaira and Hasegawa 1999). The resulting phylogenetic trees were visualized by iTOL(Letunic and Bork 2021).

### 2.3. Computational analysis

AlphaFold2 server is used to generate the structures of feruloyl esterases(Jumper et al. 2021). The molecular dynamics (MD) simulations toward the characterized FEs were performed using the GROMACS 2020.6 software package(Justin 2018, Pronk et al. 2013). The protein was solvated using the SPC water model(Zielkiewicz 2005). The protein was centered in a 10 Å cubic box with periodic boundaries. The box was filled with around 30394 water molecules. The system was neutralized by $Na^+$ and $Cl^-$ to achieve a net charge of zero. The AMBER14 force field was used for the residues of FEs (Maier et al. 2015). 5000 steps of the steepest descent followed by 5000 steps of conjugate gradients were used for energy minimization. The simulation of FEs was equilibrated by a 1 ns NVT ensemble followed by a 1 ns NPT ensemble, during which position restraints were applied to protein-heavy atoms. The production simulation was performed at 298 K for 100 ns with three replicates. The dominant conformations of FEs were obtained by clustering analysis of GROMACS with 0.1-0.25 nm cutoff. The dynamical cross-correlation matrix analysis was conducted by the previous work(Yu and Dalby 2018).

### 2.4. Deep Learning Model

To predict the promiscuity of feruloyl esterases (FEs) and their catalytic substrate spectrum, we utilized the Substrate–Product Pair-based Enzyme Promiscuity Prediction (SPEPP) model. The model utilizes transfer learning and transformer architecture to analyze enzyme-substrate relationships. It was trained on a dataset comprising experimentally verified enzyme–substrate interactions from public databases, including BRENDA. The dataset was divided into training and validation sets, and cross-validation was employed to assess the model's generalizability and prevent overfitting. Gradient-based optimization techniques were applied to fine-tune the model’s hyperparameters, with a loss function designed to balance prediction accuracy and interaction complexity. The model's capabilities were integrated into the EnzyPick web server, providing an accessible tool for enzyme screening, particularly for users without programming expertise. EnzyPick is available at http://www.biosynther.com/enzypick/(Xing et al. 2024). The

three substrates: 4-nitrophenyl ferulate, coniferyl p-coumarate, and 4-nitrophenyl feruloyl-L-arabinofuranoside were selected as probes to test enzyme promiscuity. Enzymes with a probability score exceeding 90% for all three substrates were considered potentially promiscuous FEs.

## 3. Results and Discussion

### 3.1. Enzyme similarity networks of feruloyl esterases

To explore the general characteristics of feruloyl esterases (FEs), we initially obtained all 2085 FE sequences (EC 3.1.1.73) from BRENDA database. The enzyme similarity network showed that the FE sequences were divided three main clusters (**Fig. 1**). FEs from cluster 1 and cluster 2 were predominantly derived from prokaryotes, while a subcluster (a division of main cluster) from cluster 1 was mainly from eukaryotes. Notably, the FEs from cluster 3 were primarily of eukaryotic origin. ~~Further analysis of characterized representatives revealed that 21 characterized enzymes were identified~~Through retrieving characterized FEs from the BRENDA database (EC 3.1.1.73), a total of 21 characterized FEs were identified for further analysis, with 17 representative enzymes distributed across clusters. Specifically, 9 FEs were identified in cluster 1, and 8 enzymes were found in cluster 3. Surprisingly, no characterized enzymes were distributed in cluster 2. Among all cluster 1 entities, 5 FEs (AoFE, ThFE, TaFE, TtFE and NcFE) were distributed within eukaryotic subcluster, while 4 enzymes (TwFE, TfFE, TpFE and EnFE) were distributed within prokaryotic subcluster. Interestingly, all 9 enzymes in cluster 1 were derived from prokaryotes, although they belonged to either the prokaryote subcluster or the eukaryote subcluster. Similarly, cluster 3, which represented a typical eukaryotic cluster, comprised 8 characterized enzymes (TapFE, PchFE, FuoFE, EmnFE, SccFE, RhsFE, AsnFE and ScoFE) exclusively sourced from prokaryotes. We hypothesize that there is horizontal gene transfer~~gene level transfer~~ between prokaryotic and eukaryotic clusters~~between different clusters~~. Intriguingly, upon closer examination of cluster 1 and cluster 3 using characterized representative enzymes, we observed significant differences in sequence length. FE sequences in cluster 1 were approximately 300 amino acids in length,

whereas those in cluster 3 exceeded 500 amino acids. This implies that the functional entities within cluster 1 or 3 may represent two distinct classes of FEs, each possessing unique functional characteristics.

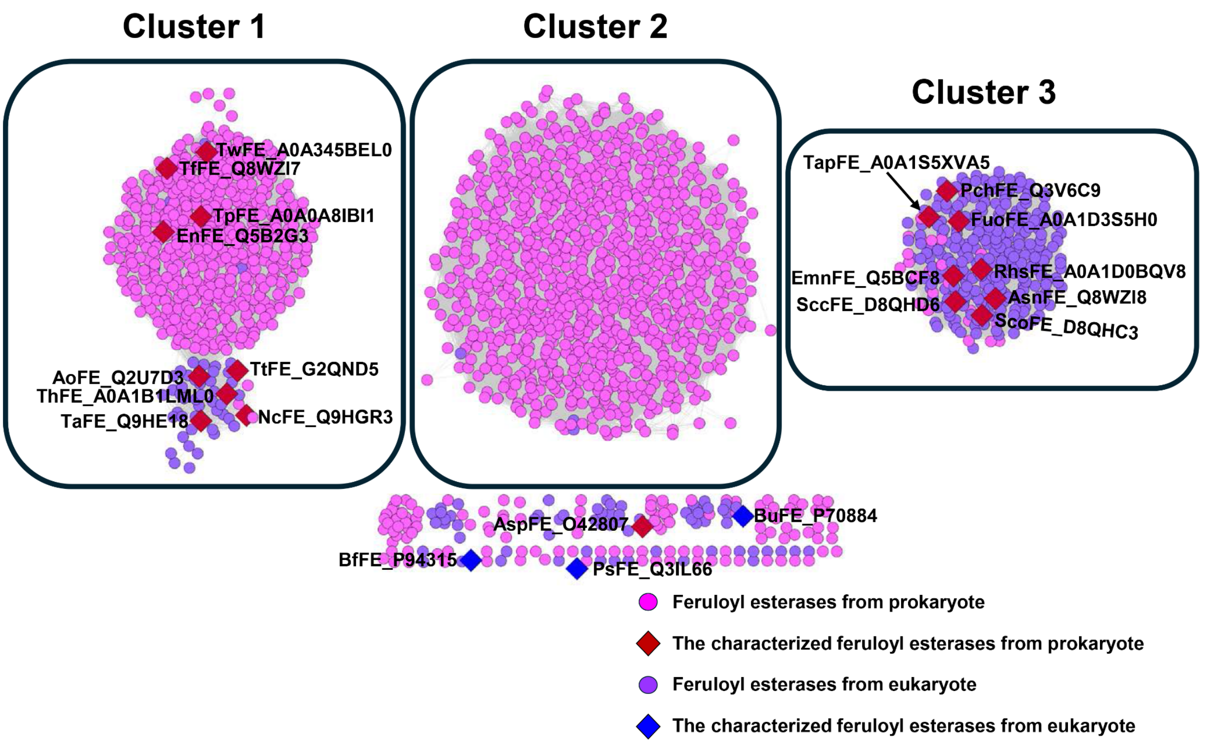


**Fig. 1** Enzyme similarity network analysis of feruloyl esterases (FEs, EC 3.1.1.73). FEs from prokaryotes are labeled pink and FEs from eukaryotes are labeled blue. The characterized FEs from prokaryotes are labeled red (diamond) and the characterized FEs from eukaryotes are labeled blue (diamond). The UniProt IDs of the characterized FEs are also labeled.

### 3.2. Phylogenetic classification of the characterized feruloyl esterases with different substrate scope

To further explore the structural and functional characteristics of FEs in clusters 1 and 3, we constructed phylogenetic trees for these two clustered FEs (**Fig. 2**). Like the enzyme similarity network, we observed that the phylogenetic tree of cluster 1 were also divided into ~~prokaryotic and eukaryotic~~two subbranches, C1_Branch 1 and C1_Branch 2. 5 representative FEs (AoFE, ThFE, TaFE, TtFE, and NcFE) were situated within the ~~eukaryotic~~ subbranch C1_Branch 1, while 4 representative enzymes (TwFE, TfFE, TpFE, and EnFE) were located within the ~~prokaryotic~~ subbranch C1_Branch 2 (**Fig. 2A**). In contrast, all 8 FEs in cluster 3 were

concentrated within two subbranches (C3_Branch 1 and C3_Branch 2) of the evolutionary tree (**Fig. 2B**). To delve deeper into the characteristics of enzymes across different branches, we gathered substrate catalytic features for all representative FEs. Surprisingly, the 5 FEs (AoFE, ThFE, TaFE, TtFE and NcFE) situated within the ~~eukaryotic~~ subbranch C1_Branch 1 exhibited a broad substrate scope in cluster 1, whereas the 4 FEs (TwFE, TfFE, TpFE and EnFE) within the ~~prokaryotic~~ subbranch C1_Branch 2 displayed a narrow substrate spectrum. Similarly, the 5 FEs (EmnFE, SccFE, RhsFE, AsnFE and ScoFE) within subbranch C3_Branch 1 showcased a broader substrate spectrum compared to the 3 FEs (TapFE, PchFE and FuoFE) within subbranch C3_Branch 2 in cluster 3 (**Table S1**). This discovery highlights the significance of understanding evolutionary divergence's influence on the functional diversity of FEs within clusters 1 and 3. Therefore, we selected the FEs with the broadest and narrowest substrate spectra in clusters 1 and 3 for further investigation (TaFE with the broadest substrate scope and TpFE with the narrowest substrate scope in cluster 1; AsnFE with the broadest substrate scope and PchFE with the narrowest substrate scope in cluster. Evolutionary divergence within these clusters of FEs is closely associated with substrate preference and functional adaptation. The FEs of broad substrate range in branches reflects adaptations to varying environmental pressures, while the FEs of narrow substrate specificity suggests more

specialized roles related to the metabolic needs of their organisms.

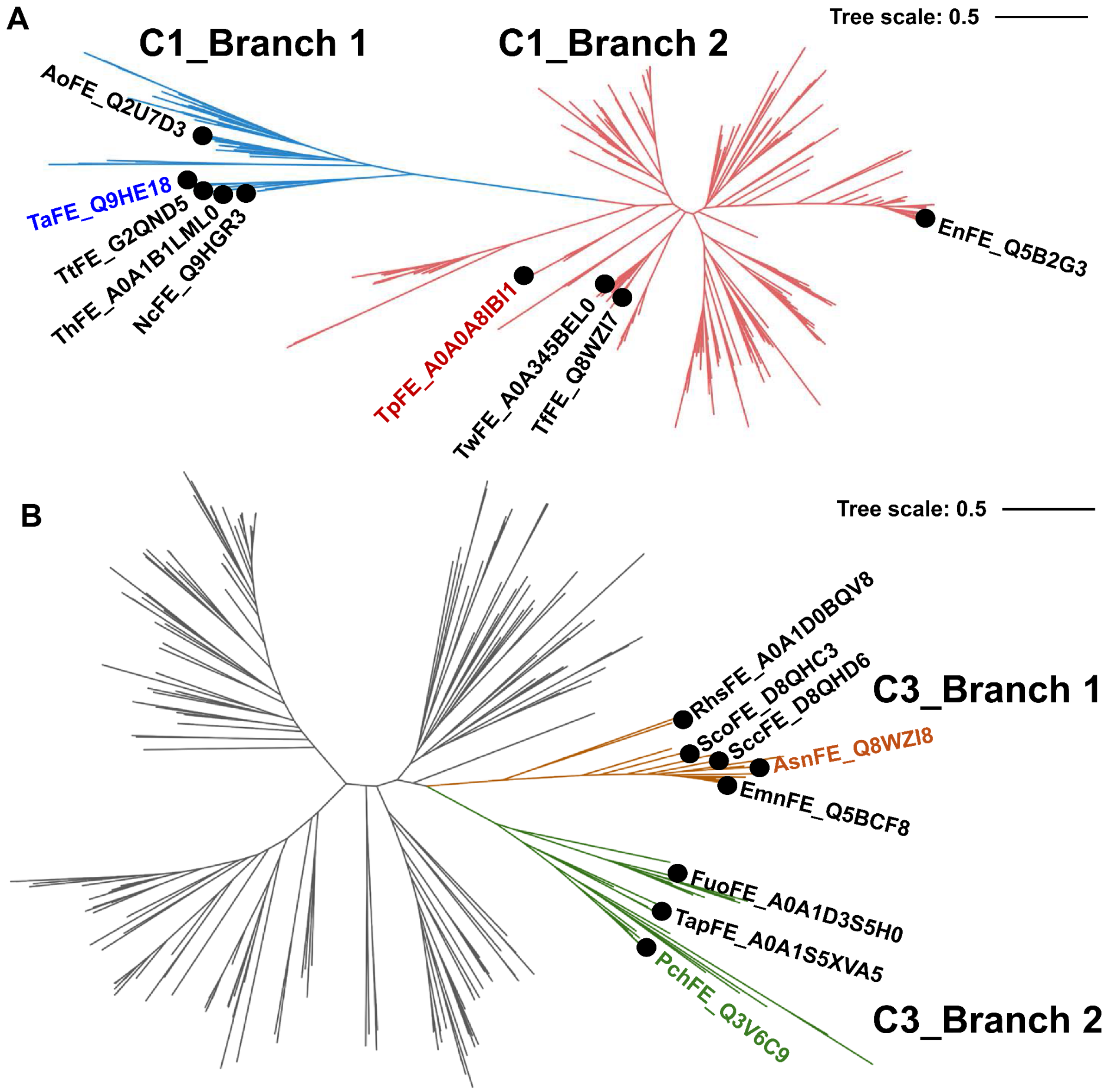


**Fig. 2** Phylogenetic tree analysis of feruloyl esterases from cluster 1 and cluster 3. A) The phylogenetic tree of feruloyl esterases from cluster 1, the characterized enzymes with different substrate scope are labeled in C1_Branch 1 (blue) and C1_Branch 2 (red). B) The phylogenetic tree of feruloyl esterases from cluster 3, the characterized enzymes with different substrate scope are also labeled in C3_Branch 1 (yellow) and C3_Branch 2 (green).

### 3.3. Structure-based for molecular dynamic characterization of feruloyl esterases with different substrate scope

TaFE and TpFE are two FEs located in cluster 1 that exhibit evolutionary divergence in enzyme similarity networks and phylogenetic trees. While TaFE exhibited a broad substrate spectrum, TpFE showed substrate specificity. To explore the correlation between the structures of these two enzymes and their catalytic features, we utilized AlphaFold2 to perform protein modeling for TaFE and TpFE, followed by structural alignment (**Fig. 3A**). We observed that both TaFE and TpFE exhibit a loose loop region and a terminal α-helix at the end of their structures, forming a "lid" structure (long loop that differ from the main structure of the protein). Structural comparison of TaFE and TpFE revealed that the lid structure in TpFE partially obstructs access to the conserved catalytic triad (Ser136, Asp220, and His276), which may contribute to its narrower substrate spectrum compared to TaFE. To further understand the dynamic information of residues of these two FEs, we conducted 100 ns molecular dynamics simulations for TaFE and TpFE, and then characterized the dynamic behaviors of each amino acids using root mean square fluctuation (RMSF) RMSF values (**Fig. S1**). We found that both TaFE and TpFE show similar RMSF values for the first 300 amino acids, indicating consistent flexibility of these amino acids. However, significant differences in flexibility were observed in the loop region and terminal α-helix after 300 amino acids, with TaFE exhibiting lower flexibility compared to TpFE (**Fig. 3B**). Hence, high flexibility and the positioning of the "lid" structure can lead to unstable protein conformation and steric hindrance to substrate access, collectively reducing substrate promiscuity. As the "lid" structure is located at the terminal end of the entire protein, we further explored the long-range correlation between the "lid" structure and the main body structures of both TaFE and TpFE. We separately calculated the dynamical cross-correlation matrices of the two FEs. Interestingly, the amino acid correlation of TaFE is much lower than that of TpFE (**Fig. 3C** and **Fig. 3D**). The high flexibility of "lid" structure of TpFE, along with the strong correlation (positive or negative) between the "lid" structure and the main body structure, may also result in an unstable protein conformation for TpFE, leading to poor substrate compatibilitypromiscuity. ConverselyOverall, the high flexibility, positioning of the lid structure, and its correlation with the main body are critical for the substrate

promiscuity of FEs.~~the stability of "lid" structure of TaFE and the weak amino acid correlation may enhance substrate compatibility of TaFE, thereby expanding its substrate catalysis range.~~

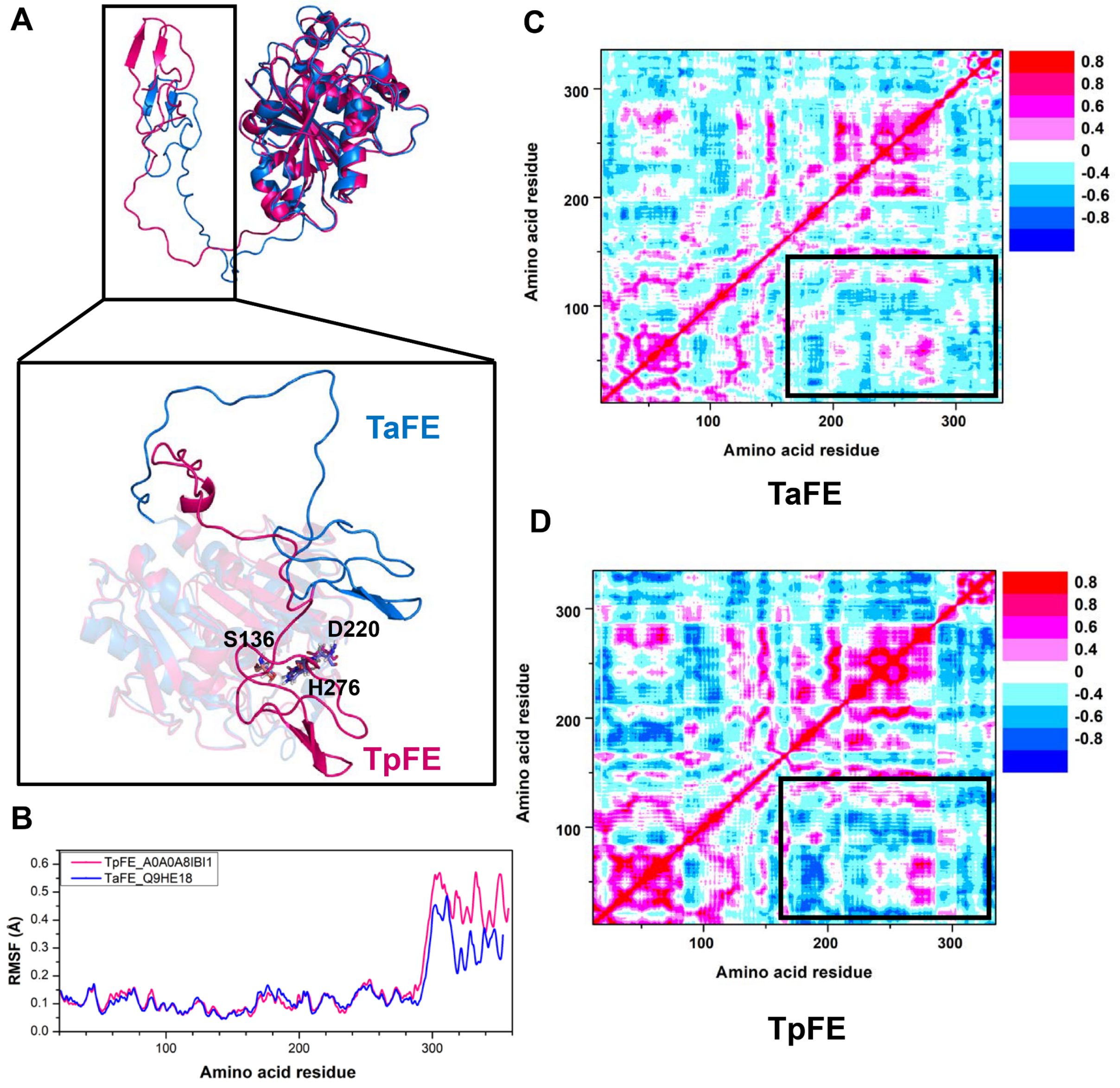


**Fig. 3** Molecular dynamics analysis of representative FEs (TpFE_A0A0A8IBI1 and TaFE_Q9HE18) with different substrate scope in cluster 1. A) The comparison of dominant conformations of TaFE (blue) and TpFE (red) with the conserved catalytic triad (Ser136, Asp220, and His276, two FEs are the same). B) The RMSF values for the α-carbon of each residue in TaFE (blue) and TpFE (red) after three parallel 100 ns MD simulations. C) and D) The dynamical cross-correlation matrix analysis of TaFE (C) and TpFE (D). The cross-correlation regions are labeled with rectangles.

To further investigate the differential characteristics of FEs in cluster 3, we analyzed two representative FEs, AsnFE and PchFE, as they exhibit different substrate scope. AsnFE

exhibited a broad substrate spectrum, while PchFE showed a narrow substrate spectrum. Similarly, we employed AlphaFold2 for protein modeling of AsnFE and PchFE, followed by 100 ns molecular dynamics simulations. By comparing RMSF values (**Fig. S2**), we observed significant differences in flexibility in two loop regions, namely Loop region 1 and Loop region 2 of AsnFE and PchFE (**Fig. 4A**). In both regions, AsnFE exhibited lower flexibility compared to PchFE, which is consistent with the results observed in FEs from cluster 1, the FEs with broader substrate spectrum usually showed lower loop flexibility (**Fig. 4A** and **Fig. 3B**). Through molecular dynamics simulations, we further obtained the dominant conformations of AsnFE and PchFE and compared their structural differences (**Fig. 4B**). By analyzing the triplet catalytic residues Ser-Asp-His, we found a higher degree of overlap between serines (S185 from AsnFE and S188 from PchFE) and histidines (H437 from AsnFE and H441 from PchFE). The catalytic residue aspartate (D397) was closer to S185 and H437 in AsnFE, whereas the catalytic residue aspartate (D407) was far from S188 and H441 in PchFE (**Fig. 4C**). Additionally, Loop region 1 of PchFE is positioned closer to the entrance of the catalytic center compared to that of AsnFE, which may hinder substrate entry into the catalytic center, resulting in a narrower substrate spectrum for PchFE (**Fig. 4D**). Since Loop region 2 of both AsnFE and PchFE are distanced from the catalytic center, Loop region 2 did not exhibit significant differences (**Fig. 4E**). ~~Consequently, we further explored the dynamic long-range correlation between the differential regions of AsnFE and PchFE and the catalytic center.~~

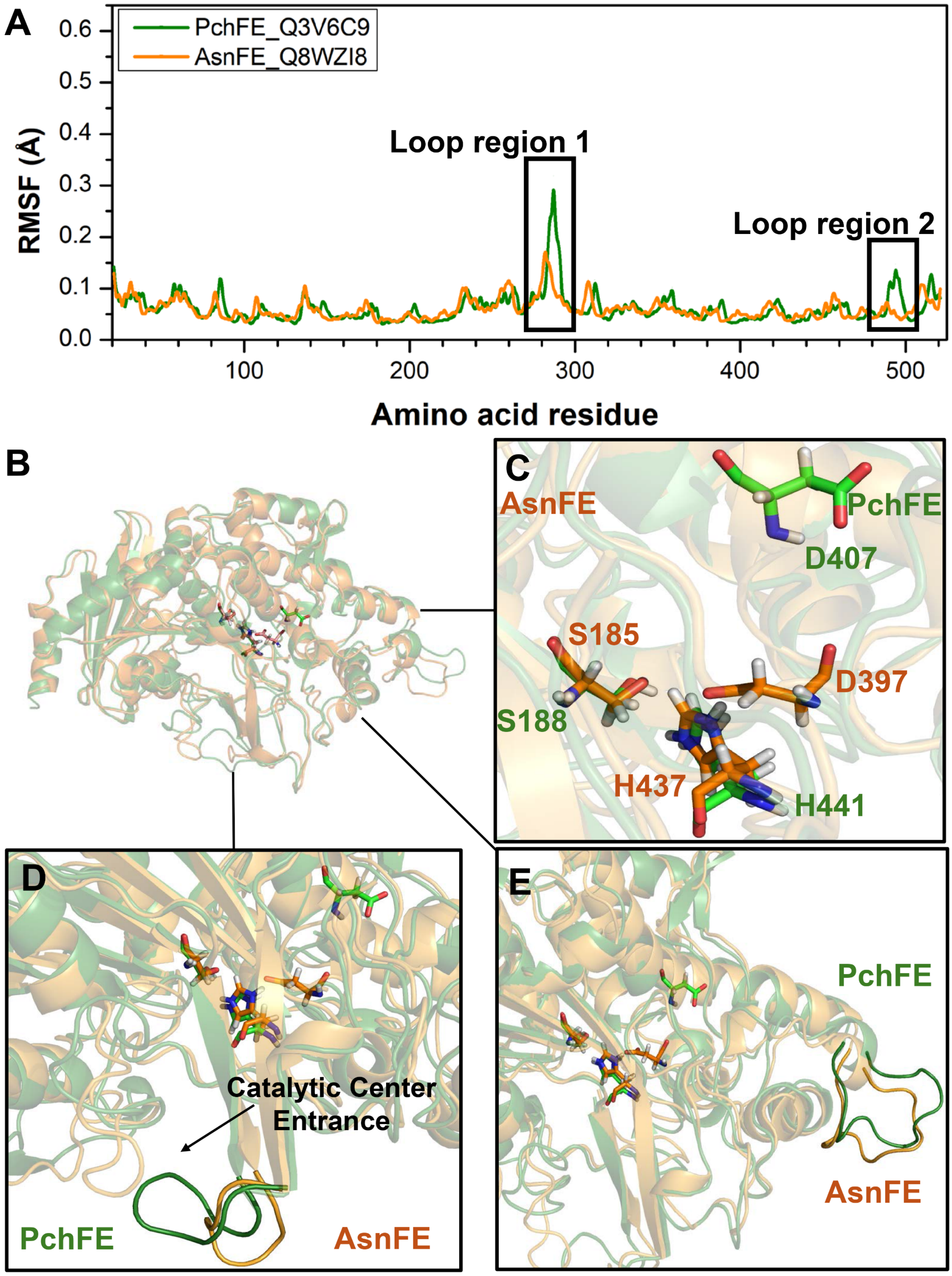


344

345 **Fig. 4** Molecular dynamics analysis of representative FEs with different substrate scope in cluster 3. A) The RMSF
346 values for the α-carbon of each residue in PchFE (green) and AsnFE (orange) after three parallel 100 ns MD
347 simulations. B) The structural alignment of dominant conformations of PchFE (green) and AsnFE (orange). The
348 triplet catalytic residues Ser-Asp-His are labeled. C) Positional relationships of the triplet catalytic residues Ser-

Asp-His in PchFE (green) and AsnFE (orange). D) Differences in the position of loop region 1 towards the catalytic center for PchFE (green) and AsnFE (orange). E) Comparison of ~~relative~~the relative positions of loop region 2 in PchFE (green) and AsnFE (orange).

We further investigated the dynamic long-range correlation between loop regions (loop region 1 and loop region 2) and the catalytic center in both AsnFE and PchFE. We separately calculated the dynamical cross-correlation matrices of these two FEs. We found that, in both AsnFE and PchFE, Loop region 2 did not exhibit significant correlation with the three catalytic residue regions. However, notably, loop region 1 of PchFE showed strong negative distant correlation with the catalytic residues serine (S188), aspartate (D407), and histidine (H441). In contrast, this distant correlation was less pronounced in AsnFE (**Fig. 5A** and **Fig. 5B**). Therefore, the high flexibility of the loop regions may propagate to the catalytic center, resulting in an unstable protein conformation for FEs and poor substrate ~~compatibility~~promiscuity. Conversely, the stability of loop structures and weak correlation with catalytically relevant residues may enhance the substrate ~~compatibility~~ promiscuity of FEs, expanding their substrate scope.

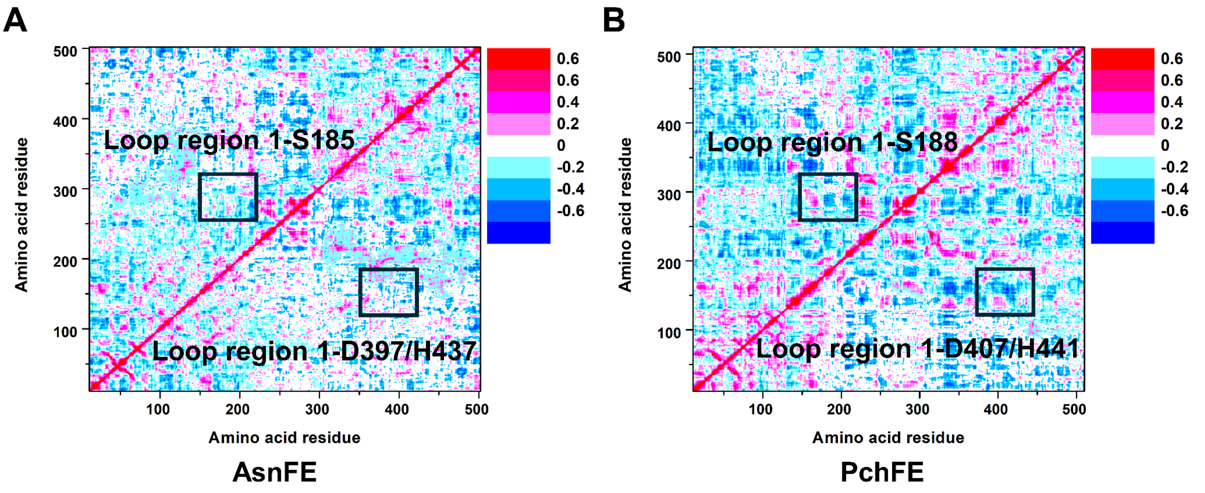


**Fig. 5** The dynamical cross-correlation matrix analysis of AsnFE (A) and PchFE (B). The cross-correlation regions are labeled with rectangles.

### 3.4. Mining of versatile feruloyl esterases using deep learning model

The use of molecular modeling and dynamic simulations can predict whether FEs belong to versatile enzymes, but this method often cannot be applied on a large scale for the prediction of a vast number of FEs. Herein, we employed the deep learning model of EnzyPick (Enzyme Selection Tool(Xing et al. 2024)) to predict potential versatile FEs (**Fig. 6**). Step 1, all FEs (3.1.1.73) were retrieved from the BRENDA database. Step 2, enzyme similarity network analysis was conducted to preliminarily cluster all FEs, and the structural or catalytic features of different clusters were determined using characterized FEs. Step 3, phylogenetic tree analysis of specific clusters was performed to locate FEs with different catalytic features, focusing on identifying FEs with broad substrate spectrum, particularly those exhibiting substrate promiscuity. Step 4, specific types of reactions were determined, and a deep learning model (such as EnzyPick) was applied to screen potential versatile FEs compatible with each reaction types. Here, we selected three reaction types, represented by the substrates 4-nitrophenyl ferulate, coniferyl p-coumarate, and 4-nitrophenyl feruloyl-L-arabinofuranoside, and predicted potential versatile FEs using the EnzyPick deep learning model. These substrates were chosen as probes to comprehensively test the enzyme promiscuity and substrate specificity of FEs, highlighting their versatility in catalyzing a wide range of ester bond hydrolysis reactions relevant to both industrial and environmental applications~~Here, we selected three reaction types and predicted potential versatile FEs using the EnzyPick deep learning model~~. The FEs with probability scores exceeding 90% in all three reaction types were selected, and 38 and 75 potential versatile FEs in clusters 1 and 3 were predicted, respectively (**Table ~~A.~~S2** and **Table ~~A.~~S3**, highlighted in yellow).

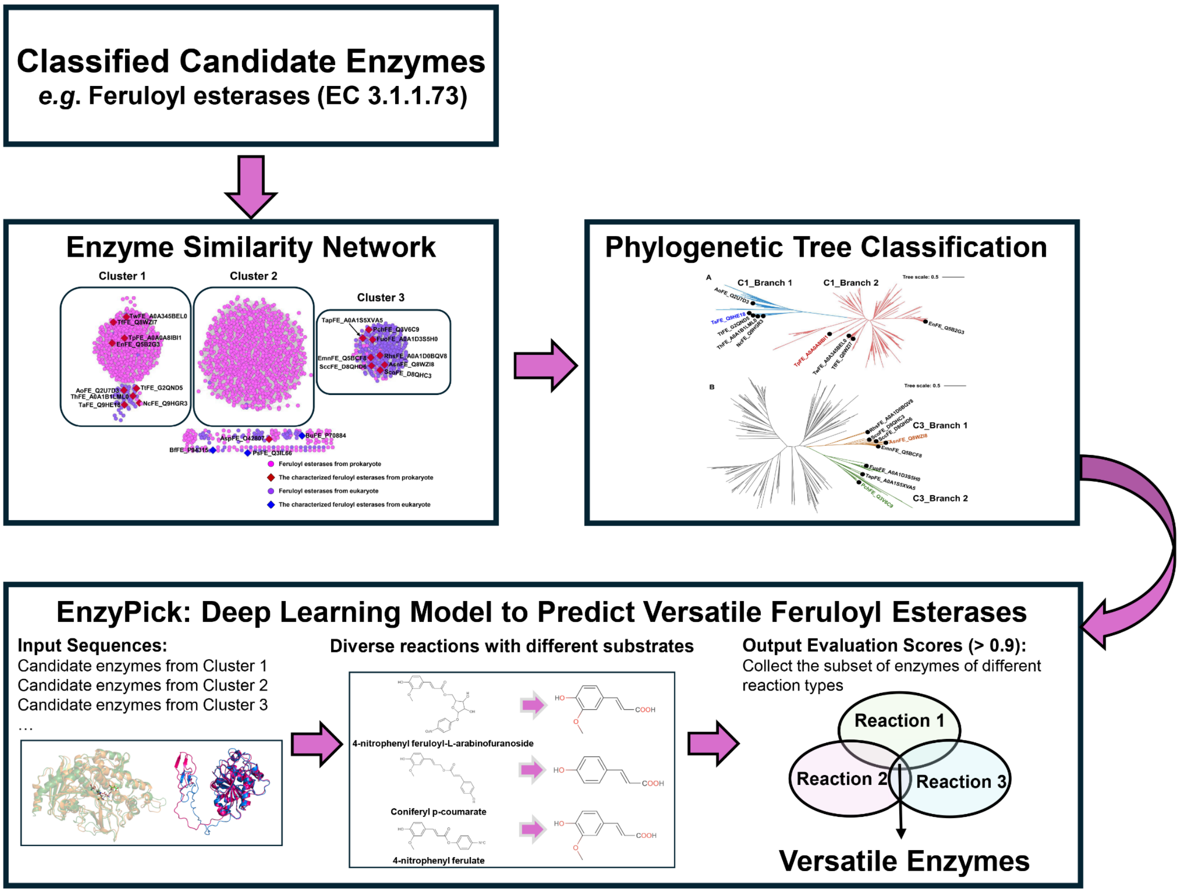


**Fig. 6** The workflow of the phylogenetic and structural feature-based deep learning strategy for mining of the versatile feruloyl esterases.

## 4. Conclusion

Feruloyl esterases (FEs, EC 3.1.1.73) play vital roles in biological synthesis and metabolism, yet identifying versatile FEs capable of catalyzing a wide range of substrates poses a challenge. This study integrated multiple methodologies to address this challenge effectively. Initially, an enzyme similarity network analysis revealed distinct clusters, with subsequent phylogenetic analysis linking phylogenetic classification with substrate promiscuity. Structural dynamics differences between promiscuous and substrate-specific FEs were explored using molecular dynamics simulations and dynamical cross-correlation matrix analysis. Additionally, the employment of deep learning models significantly expanded the repertoire of versatile FEs, identifying 38 and 75 potential candidates from cluster 1 and cluster 3, respectively, with high probability scores. The findings underscore the importance of integrating phylogenetic and

structural features with deep learning approaches for mining versatile FEs, thereby shedding light on unexplored enzymatic diversity. This holistic approach not only enhances our understanding of enzyme functionality but also expands the toolbox of biocatalysts for diverse practical applications. For instance, the identification of FEs with broad substrate specificity can drive advancements in biomass degradation for biofuel production, enabling more efficient conversion of lignocellulosic materials into bioethanol or biodiesel. Additionally, these versatile enzymes can be applied in the synthesis of value-added chemicals, such as phenolic compounds and antioxidants, which are widely used in the pharmaceutical, cosmetic, and food industries. These applications not only showcase the industrial relevance of FEs but also highlight their potential in reducing environmental impact and advancing green chemistry principles across a range of biotechnological and bioengineering processes.~~The findings underscore the importance of integrating phylogenetic and structural features with deep learning approaches for mining versatile FEs, thereby shedding light on unexplored enzymatic diversity. This holistic approach not only enhances our understanding of enzyme functionality but also expands the toolbox of biocatalysts for synthetic applications, contributing to advancements in various fields of biotechnology and bioengineering.~~

## Abbreviations

**FEs** Feruloyl esterases

**MD** Molecular dynamics

**iTOL** Interactive Tree Of Life

**SPC** Simple Point Charge

**NVT** Constant Number of particles, Volume, and Temperature

**NPT** Constant Number of particles, Pressure, and Temperature

## Declarations

### Ethics approval and consent to participate

Not applicable.

**Consent for publication**

Not applicable.

**Availability of data and materials**

The datasets generated and analysed during the current study are available in the BRENDA repository (https://www.brenda-enzymes.org/) and CAZy repository (http://www.cazy.org/) .

**Competing interests**

The authors declare that they have no competing interests.

**Acknowledgements~~Funding~~**

This work was supported by Zhejiang Shuren University Basic Scientific Research Special Funds (2024XZ010) and Zhejiang Shuren University Scientific Research Planning Project (2022R011).

**Authors' contributions**

LG designed the study, performed data analysis, and drafted the manuscript. YD contributed to data analysis and assisted with writing the manuscript. DZ contributed to data collection and partial data analysis. XP participated in the data analysis for the manuscript. XJ participated in the data collection. XY participated in the data analysis for the manuscript. YL supervised the study design, oversaw research progress, and reviewed the manuscript. All authors read and approved the final manuscript.

~~**Acknowledgements**~~

~~The authors are thankful to the laboratory of advanced technologies for conducting part of the analyses.~~

**Appendices**

List of Figures and Tables

Fig. ~~A.~~S1, Fig. ~~A.~~S2

Table ~~A.~~S1, Table ~~A.~~S2, Table ~~A.~~S3

## Reference


Akiva E, Copp JN, Tokuriki N, Babbitt PC. 2017. Evolutionary and molecular foundations of multiple contemporary functions of the nitroreductase superfamily. Proceedings of the National Academy of Sciences of the United States of America 114:E9549-E9558.

Ashok AD, Freitag JN, Irisarri I, de Vries S, de Vries J. 2024. Sequence similarity networks bear out hierarchical relationships of green cytochrome P450. Physiologia Plantarum 176 (art. e14244):11.

Bhattacharjee N, Alonso-Cotchico L, Lucas MF. 2023. Enzyme immobilization studied through molecular dynamic simulations. Frontiers in Bioengineering and Biotechnology 11 (art. 1200293):15.

Cao XT, Yang X, Xiao M, Jiang XK. 2023. Molecular Dynamics Simulations Reveal the Conformational Transition of GH33 Sialidases. International Journal of Molecular Sciences 24 (art. 6830):12.

Dallago C, Yang KK. 2023. Illuminating enzyme design using deep learning. Nature Chemistry 15:749-750.

de Oliveira DM, Finger-Teixeira A, Mota TR, Salvador VH, Moreira-Vilar FC, Molinari HBC, Mitchell RAC, Marchiosi R, Ferrarese O, dos Santos WD. 2015. Ferulic acid: a key component in grass lignocellulose recalcitrance to hydrolysis. Plant Biotechnology Journal 13:1224-1232.

Dong XY, Huang R. 2022. Ferulic acid: An extraordinarily neuroprotective phenolic acid with anti-depressive properties. Phytomedicine 105 (art. 154355):16.

Ebert MC, Pelletier JN. 2017. Computational tools for enzyme improvement: why everyone can–and should–use them. Current Opinion in Chemical Biology 37:89-96.

Gilbert J, Ermilova I, Nagao M, Swenson J, Nylander T. 2022. Effect of encapsulated protein on the dynamics of lipid sponge phase: a neutron spin echo and molecular dynamics simulation study. Nanoscale 14:6990-7002.

Gopalan N, Rodríguez-Duran LV, Saucedo-Castaneda G, Nampoothiri KM. 2015. Review on technological and scientific aspects of feruloyl esterases: A versatile enzyme for biorefining of biomass. Bioresource Technology 193:534-544.

Han JX, Liu TT, Zhang XB, Yang YQ, Shi YL, Li JT, Ma MF, Zhu WL, Gong LK, Xu ZJ. 2022. D3AI-Spike: A deep learning platform for predicting binding affinity between SARS-CoV-2 spike receptor binding domain with multiple amino acid mutations and human angiotensin-converting enzyme 2. Computers in Biology and Medicine 151 (art. 106212):8.

Jerves C, Neves RPP, Ramos MJ, da Silva S, Fernandes PA. 2021. Reaction Mechanism of the PET Degrading Enzyme PETase Studied with DFT/MM Molecular Dynamics Simulations. Acs Catalysis 11:11626-11638.

Jumper J, et al. 2021. Highly accurate protein structure prediction with AlphaFold. Nature.

Justin AL. 2018. From proteins to perturbed Hamiltonians: a suite of tutorials for the GROMACS-2018 molecular simulation package [article v1. 0]. Living Journal of Computational Molecular Science 1.

Katoh K, Rozewicki J, Yamada KD. 2019. MAFFT online service: multiple sequence alignment, interactive sequence choice and visualization. Briefings in bioinformatics 20:1160-1166.

Kerk D, Mattice JF, Valdés-Tresanco ME, Noskov SY, Ng KKS, Moorhead GB. 2021. The origin and radiation of the phosphoprotein phosphatase (PPP) enzymes of Eukaryotes. Scientific Reports 11 (art. 13681):13.

Khan KA, Memon SA, Naveed H. 2021. A hierarchical deep learning based approach for multi-functional enzyme classification. Protein Science 30:1935-1945.

Korany AH, Abouhmad A, Bakeer W, Essam T, Amin MA, Hatti-Kaul R, Dishisha T. 2020. Comparative Structural Analysis of Different Mycobacteriophage-Derived Mycolylarabinogalactan Esterases (Lysin B). Biomolecules 10 (art. 45):21.

Letunic I, Bork P. 2021. Interactive Tree Of Life (iTOL) v5: an online tool for phylogenetic tree display and annotation. Nucleic acids research 49:W293-W296.

Li D, Rui Y-x, Guo S-d, Luan F, Liu R, Zeng N. 2021. Ferulic acid: A review of its pharmacology, pharmacokinetics and derivatives. Life sciences 284:119921.

Li Dd, Zhang Z, Wang Jn, Zhang P, Liu Y, Li Yz. 2023. Estimate of the degradation potentials of cellulose, xylan, and chitin across global prokaryotic communities. Environmental Microbiology 25:397-409.

Li J, Yang J, Mu S, Shang N, Liu C, Zhu Y, Cai Y, Liu P, Lin J, Liu W. 2020. Efficient O-glycosylation of triterpenes enabled by protein engineering of plant glycosyltransferase UGT74AC1. ACS Catalysis 10:3629-3639.

Li XX, Kouzounis D, Kabel MA, de Vries RP, Dilokpimol A. 2022. Glycoside Hydrolase family 30 harbors fungal subfamilies with distinct polysaccharide specificities. New Biotechnology 67:32-41.

Maier JA, Martinez C, Kasavajhala K, Wickstrom L, Hauser KE, Simmerling C. 2015. ff14SB: improving the accuracy of protein side chain and backbone parameters from ff99SB. Journal of Chemical Theory and Computation 11:3696-3713.

Mathieu Y, Offen WA, Forget SM, Ciano L, Viborg AH, Blagova E, Henrissat B, Walton PH, Davies GJ, Brumer H. 2020. Discovery of a Fungal Copper Radical Oxidase with High Catalytic Efficiency toward 5-Hydroxymethylfurfural and Benzyl Alcohols for Bioprocessing. Acs Catalysis 10:3042-3058.

Memon SA, Khan KA, Naveed H. 2020. Enzyme Function Prediction using Deep Learning. Biophysical Journal 118:533A-533A.

Meng SQ, Li ZY, Zhang P, Contreras F, Ji Y, Schwaneberg U. 2023. Deep learning guided enzyme engineering of <i>Thermobifida fusca</i> cutinase for increased PET depolymerization. Chinese Journal of Catalysis 50:229-238.

Ming YF, Wang WK, Yin R, Zeng M, Tang L, Tang SZ, Li M. 2023. A review of enzyme design in catalytic stability by artificial intelligence. Briefings in Bioinformatics 24:19.

Oliveira DM, Mota TR, Oliva B, Segato F, Marchiosi R, Ferrarese O, Faulds CB, dos Santos WD. 2019. Feruloyl esterases: Biocatalysts to overcome biomass recalcitrance and for the production of bioactive compounds. Bioresource Technology 278:408-423.

Price MN, Dehal PS, Arkin AP. 2009. FastTree: computing large minimum evolution trees with profiles instead of a distance matrix. Molecular biology and evolution 26:1641-1650.

Pronk S, et al. 2013. GROMACS 4.5: a high-throughput and highly parallel open source molecular simulation toolkit. Bioinformatics 29:845-854.

Raj ND, Singh D. 2022. A critical appraisal on ferulic acid: Biological profile, biopharmaceutical challenges and nano formulations. Health Sciences Review 5:100063.

Shi L, Xiong Q, Ao FK, Wan TY, Xiao XJ, Liu XY, Sun BQ, Tungtrongchitr A, Leung TF, Tsui SKW. 2023. Comparative analysis of cysteine proteases reveals gene family evolution of the group 1 allergens in astigmatic mites. Clinical and Translational Allergy 13 (art. e12324):12.
Shimodaira H, Hasegawa M. 1999. Multiple comparisons of log-likelihoods with applications to phylogenetic inference. Molecular biology and evolution 16:1114.
Shu YX, Hai YR, Cao LH, Wu JM. 2023. Deep-learning based approach to identify substrates of human E3 ubiquitin ligases and deubiquitinases. Computational and Structural Biotechnology Journal 21:1014-1021.
Shukla D, Nandi NK, Singh B, Singh A, Kumar B, Narang RK, Singh C. 2022. Ferulic acid-loaded drug delivery systems for biomedical applications. Journal of Drug Delivery Science and Technology 75 (art. 103621):21.
Tao ZY, Dong BZ, Teng ZX, Zhao YM. 2020. The Classification of Enzymes by Deep Learning. Ieee Access 8:89802-89811.
Wang YQ, Xing ST, Zhao XY, Chen X, Zhan CG. 2024. Unraveling the allosteric mechanisms of prolyl endopeptidases for celiac disease therapy: Insights from molecular dynamics simulations. International Journal of Biological Macromolecules 259 (art. 129313):11.
Wittmund M, Cadet F, Davari MD. 2022. Learning Epistasis and Residue Coevolution Patterns: Current Trends and Future Perspectives for Advancing Enzyme Engineering. Acs Catalysis 12:14243-14263.
Xing H, Cai P, Liu D, Han M, Liu J, Le Y, Zhang D, Hu Q-N. 2024. High-throughput prediction of enzyme promiscuity based on substrate–product pairs. Briefings in bioinformatics 25:bbae089.
Yu H, Dalby PA. 2018. Exploiting correlated molecular-dynamics networks to counteract enzyme activity–stability trade-off. Proceedings of the National Academy of Sciences 115:E12192-E12200.
Zaboli M, Saeidnia F, Zaboli M, Torkzadeh-Mahani M. 2021. Stabilization of recombinant D-Lactate dehydrogenase enzyme with trehalose: Response surface methodology and molecular dynamics simulation study. Process Biochemistry 101:26-35.
Zallot R, Oberg N, Gerlt JA. 2019. The EFI web resource for genomic enzymology tools: leveraging protein, genome, and metagenome databases to discover novel enzymes and metabolic pathways. Biochemistry 58:4169-4182.
Zhai YM, Wang TY, Fu YM, Yu T, Ding Y, Nie HG. 2023. Ferulic Acid: A Review of Pharmacology, Toxicology, and Therapeutic Effects on Pulmonary Diseases. International Journal of Molecular Sciences 24 (art. 8011):20.
Zhang L-J, Wang D-G, Zhang P, Wu C, Li Y-Z. 2022a. Promiscuity characteristics of versatile plant glycosyltransferases for natural product glycodiversification. ACS Synthetic Biology 11:812-819.
Zhang P, Ji Y, Meng S, Li Z, Hirtz D, Elling L, Schwaneberg U. 2023. A Phylogeny-based Directed Evolution Approach to Boost the Synthetic Applications of Glycosyltransferases. Green Chemistry.
Zhang P, Meng S, Li Z, Hirtz D, Elling L, Zhu L, Ji Y, Schwaneberg U. 2024. A comparative molecular dynamics approach guides the tailoring of glycosyltransferases to meet synthetic applications. Green Chemistry 26:9186-9194.
Zhang P, Zhang L, Jiang X, Diao X-t, Li S, Li D-d, Zhang Z, Fang J, Tang Y-j, Wu D-l. 2022b. Docking-guided rational engineering of a macrolide glycosyltransferase glycodiversifies epothilone B. Communications biology 5:100.
Zhang P, Zhang Z, Zhang L, Wang J, Wu C. 2020. Glycosyltransferase GT1 family: Phylogenetic distribution, substrates coverage, and representative structural features. Computational and structural biotechnology journal 18:1383-1390.

Zheng M, Liu Y, Zhang G, Yang Z, Xu W, Chen Q. 2024. The antioxidant properties, metabolism, application and mechanism of ferulic acid in medicine, food, cosmetics, livestock and poultry. Antioxidants 13:853.
Zielkiewicz J. 2005. Structural properties of water: comparison of the SPC, SPCE, TIP4P, and TIP5P models of water. Journal Of Chemical Physics 123:104501.
