# Supplementary material for: Mining versatile feruloyl esterases: phylogenetic classification, structural features, and deep learning model
Source: Bioresour Bioprocess. 2025 Jan 29;12(1):7. doi: 10.1186/s40643-024-00835-8 (PMC11780051; doi:10.1186/s40643-024-00835-8)
Supplement: Supplementary file 2 — Supplementary Material 2 [file 40643_2024_835_MOESM2_ESM.docx]

**Mining Versatile Feruloyl Esterases: Phylogenetic Classification, Structural Features, and Deep Learning Model**

Liang Guo^1^, Yuxin Dong^2^, Deyong Zhang^1^, Xinrong Pan^1^, Xinjie Jin^3^, Xinyu Yan^4^, Yin Lu^1^*

1. Key Laboratory of Pollution Exposure and Health Intervention of Zhejiang Province, College of Biological and Environment Engineering, Zhejiang Shuren University, Hangzhou 310015, China

2. Jinan No.1 High School, Jinan 250014, China

3. College of Life and Environmental Science, Wenzhou University, Wenzhou 325035, Zhejiang, China

4. College of Agriculture, Yangtze University, Jingzhou 434000, Hubei Province, China

*Corresponding authors

Yin Lu (luyin@zjsru.edu.cn)

# 1. Additional Figures

##



## Fig. S1 The RMSF values for the α-carbon of each residue of TpFE and TaFE with three parallel MD simulations.





## Fig. S2 The RMSF values for the α-carbon of each residue of PchFE and AsnFE with three parallel MD simulations.
